# Dual targeting of AMRC12 and *Malassezia globosa* disrupts MYC liquid condensates-driven nuclear pore complex biogenesis in neuroblastoma

**DOI:** 10.7150/thno.120935

**Published:** 2026-01-01

**Authors:** Anpei Hu, Chunhui Yang, Zhijie Wang, Xiaolin Wang, Xinyue Li, Shunchen Zhou, Bosen Zhao, Jiaying Qu, Xiaojing Wang, Liduan Zheng, Qiangsong Tong

**Affiliations:** 1 Department of Pediatric Surgery, Union Hospital, Tongji Medical College, Huazhong University of Science and Technology, 1277 Jiefang Avenue, Wuhan 430022, Hubei Province, P. R. China.; 2 Department of Pathology, Union Hospital, Tongji Medical College, Huazhong University of Science and Technology, 1277 Jiefang Avenue, Wuhan 430022, Hubei Province, P. R. China.; 3 Department of Geriatrics, Union Hospital, Tongji Medical College, Huazhong University of Science and Technology, 1277 Jiefang Avenue, Wuhan 430022, Hubei Province, P. R. China.

**Keywords:** neuroblastoma, armadillo repeat containing 12, MYC, *Malassezia globosa*, nuclear pore complex

## Abstract

**Rationale:** Neuroblastoma (NB) is a predominant extra-cranial malignancy in childhood, while molecular drivers of its progression and effective treatment strategies have yet to be clarified.

**Methods:** RNA sequencing was performed to identify transcriptional regulators and corresponding target genes. To explore the biological effects and underlying mechanisms of these regulators, a comprehensive methodology was utilized, encompassing chromatin immunoprecipitation, dual-luciferase reporter assay, qRT-PCR, western blot, alongside gene over-expression and silencing techniques, co-immunoprecipitation, and mass spectrometry. The MTT assay, soft agar colony formation, Matrigel invasion, and nude mouse xenograft models were applied to assess oncogenic properties. Patient survival was analyzed using the log-rank test.

**Results:** Armadillo repeat containing 12 (ARMC12) was identified as a MYC-interacting modulator within liquid condensates to up-regulate critical nucleoporin-encoding targets (*NUP62*/*NUP93*/*NUP98*), which promoted nuclear pore complex (NPC) biogenesis to facilitate nuclear trafficking of oncogenic effectors, thereby enhancing invasion and metastasis of NB. As a protein within extracellular vesicles of *Malassezia globosa* colonizing NB tissues, MGL_0381 also facilitated MYC transactivation via physical interaction to accelerate NPC biogenesis and NB progression. Tioconazole (TCZ) and UU-T02 were identified as efficient inhibitors blocking ARMC12-MYC and MGL_0381-MYC interaction, and synergistically reduced NPC number and aggressive features of NB. High *ARMC12*, *MYC*, *NUP62*, *NUP93*, or *NUP98* levels served as markers of unfavorable patient outcomes in clinical cohorts.

**Conclusions:** These findings collectively demonstrate that dual targeting of AMRC12 and *Malassezia globosa* disrupts MYC liquid condensates-driven NPC biogenesis during NB progression.

## Introduction

Neuroblastoma (NB), an extracranial malignancy originating from sympathetic nervous system, is most prevalent in pediatric populations, and presents heterogeneous clinical behavior varying from spontaneous regression to aggressive metastatic dissemination 1. Despite multimodal therapeutic strategies incorporating surgical resection, cytotoxic chemotherapy, and radiation therapy, over 50% of high-risk NB patients suffer from rapid recurrence, local invasion, or systemic metastasis, culminating in dismal 5-year survival rates below 20% 1, 2. Therefore, it is urgent to delineate the molecular drivers and develop precision therapeutics targeting aggressive behaviors of NB. Beyond the well-known oncogenic driver *MYCN* that is amplified in almost 20% of high-risk NB, elevated *MYC* expression is an independent marker and defines a distinct high-risk subset in approximately 10% of cases 3, while its roles and protein partners in NB progression require systematic interrogation.

Nuclear pore complex (NPC), a critical gateway for nucleocytoplasmic transport, contributes to malignant transformation and tumor progression via facilitating karyopherin β superfamily-mediated transport of macromolecules 4. Certain proteins (such as β-catenin) interact directly with NPC components for nuclear translocation 5, 6. Many cancer cells exhibit increase of NPC numbers and addiction on nuclear transport machinery 4. The NPC is consisted of 30 nucleoporins (NUPs) to form distinct structural modules, including central cylinder, cytoplasmic and nuclear rings, cytoplasmic filaments, and nuclear basket 7. NPC biogenesis is a highly ordered process involving sequential recruitment of NUP107-160 (early assembly) and NUP93-205 (later assembly) complexes 8. Some NUP components, such as NUP62, NUP93, and NUP98, are linked to tumorigenesis and aggressiveness 5, 6, 9, 10. *NUP62* is up-regulated in renal clear cell carcinoma, glioma, or adrenocortical carcinoma, and correlated with poor prognosis of patients 11. As a critical NUP over-expressing in metastatic hepatocellular carcinoma 6, breast cancer 12, and cervical cancer 13, *NUP93* facilitates the proliferation and metastasis of cancer cells by mediating nuclear translocation of β-catenin 5, 6. NUP98, a NUP localizing at both cytoplasmic and nucleoplasmic sides of NPC, contributes to progression of acute myeloid leukemia via recurrent chromosomal translocations 14. Meanwhile, reducing NPC numbers is sufficient to induce regression of melanoma and colorectal cancer via affecting nuclear transport, gene expression, or DNA damage repair 4. These findings underscore NPC components as pivotal regulators and therapeutic targets of tumorigenesis. Nonetheless, the contributions of NUPs and regulation of their expression in the context of NB progression remain elusive.

In the current study, through integrating proteomic profiling, chromatin immunoprecipitation sequencing (ChIP-seq), and transcriptome-wide characterization, we discover that armadillo repeat containing 12 (ARMC12) serves as a co-factor of transcription factor MYC to drive the expression of nucleoporin-encoding genes (*NUP62*, *NUP93*, and *NUP98*), leading to elevation in NPC number and nuclear translocation of transcriptional regulators, such as AMRC12 15, MYC 16, cut like homeobox 1 (CUX1) 17, or E2F transcription factor 1 (E2F1) 18, and invasion or metastatic dissemination of NB cells. In addition, as a protein within extracellular vesicles (EVs) of *Malassezia globosa* (*M. globosa*) colonizing NB tissues, MGL_0381 interacts with and facilitates MYC transactivation to drive NPC biogenesis and NB progression. Through molecular docking and affinity purification assays, tioconazole (TCZ) and UU-T02 are identified as efficient inhibitors blocking ARMC12-MYC or MGL_0381-MYC interaction. Administration of both TCZ and UU-T02 exerts synergetic inhibitory effects on NPC biogenesis and aggressiveness of NB, highlighting the biological significance of MGL_0381/*MYC* and *ARMC12*/*MYC* axes in orchestrating NB malignant progression.

## Materials and Methods

### Cell lines and culture

Following acquisition from the American Type Culture Collection (ATCC, Rockville, MD) or European Collection of Authenticated Cell Cultures (ECACC, Salisbury, UK), the employed human cell lines, HEK-293T (CRL-3216), SH-SY5Y (CRL-2266), SK-N-AS (CRL-2137), SK-N-BE(2) (CRL-2271), SH-EP (CVCL_0524), underwent authentication via short tandem repeat profiling. Subsequent functional experiments were conducted within six months after cell thawing. Routine screening for mycoplasma contamination was conducted with the MycoAlert^®^ PLUS Kit (Takara, Japan). Specifically, cells validated to be free of contamination were grown in a humidified incubator at 37 °C with 5% CO₂, using DMEM or RPMI-1640 medium fortified with 10% fetal bovine serum (FBS, Thermo Fisher Scientific, Gaithersburg, MD).

### Isolation and quantitative analysis of RNA

Total RNA was extracted using the RNeasy Mini Kit (Qiagen, Germany), which was then reversely transcribed into cDNA with the ProtoScript^®^ II kit (New England Biolabs, Ipswich, MA). Subsequent quantitative PCR (qPCR) was carried out using SYBR Green PCR Master Mix (Takara, Japan) and sequence-specific primers (listed in Table S1). The mRNA quantification was calculated by 2 ^-△△Ct^ method 16, 18-23.

### Western blotting

Following lysis of tissues or cultured cells with RIPA buffer (Promega, Madison, WI), the resulting protein extracts were analyzed by western blotting using established methodologies 16, 18-23 and antibodies specific against ARMC12 (PA5-111006, Thermo Fisher Scientific), MYC (ab32072), NUP62 (ab188413), NUP93 (ab53750), NUP98 (ab124980), CUX1 (ab307821), E2F1 (ab4070), histone H3 (HH3, ab5103), glyceraldehyde-3-phosphate dehydrogenase (GAPDH, ab8245), glutathione S-transferase (GST, ab307273), His-tag (ab18184), hemagglutinin (HA)-tag (ab236632), FLAG-tag (ab125243), or β-actin (ab7291, Abcam Inc., Cambridge, MA).

### Assay for dual-luciferase reporter activity

Amplicons of human *NUP62* (-791/+134), *NUP93* (-1000/+197), or *NUP98* (-704/+146) promoter regions were generated from genomic DNA with primers detailed in Table S2 and cloned into pGL3-Basic vector (Promega). The MYC-binding motifs were mutagenized using the Q5^®^ system (New England Biolabs) with custom-designed primers (Table S2). For analyzing MYC transactivation, luciferase reporter constructs were generated through ligating oligonucleotide pairs with three typical MYC response elements (Table S2) into pGL4.1 luciferase vector (Promega). The transcriptional activity was measured via LiveCell™ Dual-Luciferase Assay Kit (Biotium, Fremont, CA) and a GloMax^®^ Luminescence Reader (Promega) 16, 18-23.

### Chromatin immunoprecipitation (ChIP) assay

ChIP experiments were conducted using the EZ-ChIP kit (Millipore, Burlington, MA) 16, 18-23, with antibodies targeting MYC (ab32072). For real-time qPCR analysis, immunoprecipitated DNA was amplified using SYBR Green PCR Master Mix (Takara) and primers listed in Table S1. Isotype IgG served as the negative control for data normalization.

### Modulation of gene expression

The coding sequence of human *ARMC12* (1104 bp) or *MYC* (1368 bp) was amplified from NB specimens (Table S2), while their full-length fragments or truncations were inserted into pCMV-3Tag-1A (Addgene, Cambridge, MA), pCMV-HA (Addgene), pGEX-6P-1 (Beyotime Biotechnology, Haimen, China), pET28-A (Addgene) 15, 16, pET28a-mCherry (Genprice Inc., San Jose, CA), pET28a-EGFP (Genprice Inc.), pCMV-N-MYC (Addgene), or CV186 (Genechem Co., Ltd, Shanghai, China). The NLS-mCherry-NES reporter (pDN160) was obtained from Addgene. The Q5^®^ system (New England Biolabs) was utilized to generate mutations at specified sites, using primers provided in Table S2. To generate stable cell lines, short hairpin RNA (shRNA) or small guide RNA (sgRNA) oligonucleotides (Table S3) were first cloned into GV298 (Genechem), dCas9-VPR, or dCas9-BFP-KRAB (Addgene) backbones, which was followed by antibiotic selection using neomycin or puromycin (Thermo Fisher Scientific).

### Gene expression restoration

The *ARMC12* expression vector was used to transfect tumor cells in order to prevent gene expression affected by *MYC* knockdown. The Genesilencer reagent (Genlantis, San Diego, CA) was used to transfect shRNAs targeting *ARMC12* (Table S3) to rescue *MYC* over-expression-induced changes in target gene expression. As controls, an empty vector or scramble shRNA (sh-Scb) was used (Table S3).

### Lentivirus production

To produce lentivirus, HEK-293T cells were co-transfected with the transfer vector and packaging plasmids psPAX2 and pMD2G (Addgene). Viral supernatants were harvested at 36 and 60 h post-transfection. Following filtration through 0.45 μm low-protein-binding polyvinylidene fluoride (PVDF) membranes (Millipore), the lentiviral particles were concentrated by ultracentrifugation (120,000 × g, 2 h, 4 °C) and finally resuspended in phosphate-buffered saline (PBS) at 1/100 of the original volume 16, 18-20.

### RNA sequencing (RNA-seq)

From aliquots of 1 × 10⁶ cells, total RNA was purified employing the RNeasy Mini Kit (Qiagen) for subsequent RNA sequencing. The RNA-seq library was constructed by Wuhan SeqHealth Technology Co., Ltd., in accordance with standard Illumina protocols and sequenced on the HiSeq X Ten platform (Illumina), yielding 100-bp paired-end reads. Subsequent data processing involved quantification of gene-level reads with HTSeq (version 0.6.0) under the union-counting mode, followed by normalization using fragments per kilobase of transcript per million mapped reads (FPKM). The raw and processed data were publicly accessible through the Gene Expression Omnibus (GEO) repository under the accession GSE107516.

### Co-immunoprecipitation (co-IP) and mass spectrometry

Following co-IP with specific antibodies for ARMC12 (PA5-111006, Thermo Fisher Scientific), MYC (ab32072), FLAG-tag (ab125243), or HA-tag (ab236632, Abcam Inc., Cambridge, MA), the bead-bound protein complexes were eluted. These eluates were then subjected to sodium dodecyl sulfate (SDS)-polyacrylamide gel electrophoresis (PAGE) separation and analyzed either by Coomassie blue staining/immunoblotting or by mass spectrometry (Wuhan SpecAlly Life Technology Co., Ltd., China) 16, 18-23.

### Bimolecular fluorescence complementation (BiFC)

The split-Venus system was constructed by inserting *ARMC12* (1104 bp) or *MYC* (1368 bp) cDNA into pBiFC vectors (Addgene) followed by co-transfection into tumor cells with Lipofectamine 2000 (Invitrogen) for 24 h. Following this, cells were incubated in standard medium at 37 °C with 5% CO₂ for 10 h to allow fluorophore maturation. Venus reconstitution was monitored by confocal microscopy with the following settings: excitation at 488 nm and emission collected between 500-550 nm 16, 18-20.

### Immunofluorescence staining

For paraffin-embedded tissues, the sections were subjective to deparaffinization and rehydration, and blocked with 5% normal serum to prevent non-specific binding. Cultured cells were seeded onto coverslips, fixed with 4% paraformaldehyde for 10 min, and blocked with 5% milk for 1 h. Following this, samples were subjected to incubation with the respective primary antibodies targeting ARMC12 (PA5-111006, Thermo Fisher Scientific, 1 : 200), MYC (ab32072, Abcam Inc., 1 : 200), nuclear pore complex (mAb414, ab24609, Abcam Inc., 1 : 200), or Lamin A (ab108595, Abcam Inc., 1 : 200) overnight at 4 °C. After washing, samples on coverslips were stained sequentially with secondary antibodies (Alexa Fluor^®^ 488 or 594, Cell Signaling Technology Inc. Danvers, MA) and 4′,6-diamidino-2-phenylindole (DAPI, Sigma, St. Louis, MO), prior to image acquisition on a Nikon confocal microscope 16, 18-20.

### 18S rRNA sequencing

Tissue homogenates in PBS (500 μL) were mechanically lysed via vortex-sonication followed by Proteinase K digestion (2.5 μg/mL; Thermo Fisher Scientific) at 55 °C overnight. DNA was extracted using the DNeasy PowerSoil Pro system (Qiagen), quantified via Qubit BR assay (Thermo Fisher Scientific), and processed for Internal Transcribed Spacer (ITS) sequencing (Wuhan SeqHealth Technology Co., Ltd.).

### Fungal fluorescence *in situ* hybridization (FISH)

Fungal colonization was localized using a Cy3-conjugated 18S rRNA probe (Table S1; λex 561 nm/λem 570 nm; Servicebio Tech, China) through FISH. Paraffin-embedded tissue sections (4 μm) underwent sequential blocking with hybridization buffer (37 °C, 30 min), probe hybridization (20 μM in buffer, 37 °C, overnight), and stringent washes. Nuclear counterstaining with DAPI preceded microscopic analysis using a Nikon Eclipse Ti system 24.

### *M. globosa* culture and MalaEx preparation

*M. globosa* (MYA-4612, ATCC) was cultured on Modified Leeming and Notman agar medium (mLNA, ATCC) plates modified to contain 0.5% Tween 60, 1% glycerol, and 2% olive oil at 32 °C 25. MalaEx was prepared via continuous ultrafast centrifugation from culture supernatant of *M. globosa* for 48 h. Briefly, culture supernatants underwent sequential centrifugation (1,200 g × 5 min, 3,000 g × 30 min) with intermediate pellet removal. Clarified samples were sterile-filtered (0.22 μm, Merck, Germany) followed by ultracentrifugation steps (10,000 g × 30 min, 100,000 g × 90 min × 2 cycles) with PBS washing. Final exosome pellets were reconstituted in 100 μl PBS. MalaEx was characterized through morphological analysis and nanoparticle tracking, with subsequent proteomic profiling via liquid chromatography-tandem mass spectrometry (LC-MS/MS), which was performed by SpecAlly Life Technology Co., Ltd in Wuhan, China.

### Mass spectrometry

Proteomic analysis was performed using a Q Exactive Plus mass spectrometer (Thermo Fisher Scientific) coupled to an EASY-nLC 1200 nanoflow liquid chromatography system. For peptide separation, a C18 analytical column (Acclaim PepMap; 75 μm × 150 mm, 2 μm, 120 Å) was used with a 30-min linear gradient from 0.1% formic acid to 80% acetonitrile/0.1% formic acid, at a constant flow rate of 300 nL/min. Data-dependent acquisition (DDA) parameters included: full MS scans [70 K resolution, 3 × 10⁶ automatic gain control (AGC), 20 ms max injection time, m/z 350-1800) followed by top-15 HCD-MS/MS (resolution at 17,500, AGC target of 2 × 10^5^, maximum injection time of 100 ms, and normalized collision energy of 28%) with 1.6 Da isolation window and 35-s dynamic exclusion. Raw files were processed through MaxQuant (v1.6.6) against the UniProt Human database, applying 1% false discovery rate (FDR) thresholds at peptide and protein levels.

### Monitoring *in vitro* phase separation

Recombinant His-tagged proteins were expressed by transforming *ARMC12*/*MYC* constructs into BL21 *E. coli* (Thermo Fisher Scientific) and purified using His-tag affinity chromatography (Thermo Fisher Scientific). The efficiency of protein expression and purification was evaluated by SDS-PAGE followed by Coomassie blue staining, in conjunction with quantitative analysis using ImageJ (https://imagej.nih.gov/ij) to determine sample purity. Phase separation assays were conducted using glass-bottomed dishes. For droplet formation assays, 40 μM protein solutions in buffer containing 10% glycerol, 50 mM Tris-HCl (pH 7.5), and 1.0 mM dithiothreitol were mixed with 10% polyethylene glycol-8000 and imaged with an oil-immersion Olympus FV3000 confocal system 19.

### Phase separation in live cells

Cells expressing mCherry-fused proteins or endogenous targets were cultured on glass-bottom dishes (MatTek, Ashland, MA), with nuclear counterstained by DAPI (Thermo Fisher Scientific) for 10 min. Confocal microscopy (Olympus FV3000) was performed after PBS washes. Phase separation puncta (> 0.5 μm diameter) were analyzed 19.

### Fluorescence recovery after photobleaching (FRAP)

The Olympus FV3000 confocal laser scanning microscope was employed to perform FRAP assays *in vitro*. Target droplets were photobleached for 10 s using alternating 488 nm and 561 nm laser pulses at 50% maximum power, with simultaneous time-lapse imaging (1 frame/s) to monitor fluorescence recovery. All imaging was conducted under controlled environmental conditions (37 °C, 5% CO₂). FRAP assay was conducted in live cells using a climate-controlled chamber. Target regions were bleached (488/561 nm lasers, 50% power, 5 s) and imaged at 500 ms intervals for 60 s (Olympus FV3000, 60 × oil objective). Following background subtraction, the fluorescence intensities were measured and normalized to the average pre-bleach level. All quantifications were performed with FIJI/ImageJ software (https://imagej.nih.gov/ij) 19.

### Transmission electron microscopy (TEM)

TEM was performed on tumor cells cultivated in 10-cm dishes and exposed to 2.5% glutaraldehyde. After fixation with 1% osmic acid for 2-3 h, the cells were dehydrated and paraffin-embedded. Ultrathin sections (70 nm) were then contrasted with uranyl acetate and lead citrate prior to observation under a transmission electron microscope (Delong America Inc., Quebec, CA).

### Affinity purification

His-tagged MGL_0381 was provided by ChinaPeptides (Shanghai, China). The NHS magenetic beads (HY-K0227, MedChemExpress, Monmouth Junction, NJ) were incubated with 20 mM TCZ or UU-T02 in N,N-dimethylformamide. Following a 2-h incubation at 4 °C with recombinant proteins or nuclear extracts, beads pre-loaded with TCZ or UU-T02 (0.5 mg) were collected. Subsequently, bound proteins were eluted using a lysis buffer containing 0.5% NP-40. Finally, the eluates were analyzed by either immunoblotting or mass spectrometry 26.

### Differential scanning fluorimetry (DSF)

Recombinant MYC (GST-tagged) or ARMC12 (His-tagged) proteins were combined with SYPRO Orange dye (Invitrogen) in PBS buffer. The mixture was subjected to a thermal ramp from 40 °C to 90 °C at a rate of 1 °C per minute, with fluorescence intensity monitored at each degree. The resulting data (fluorescence vs. temperature) were fitted to a Boltzmann model for determining the protein melting temperature (Tm) 27.

### Cellular viability assay

Viability of cells was determined by the MTT assay (Sigma, a colorimetric method) with absorbance readings taken at 570 nm (reference wavelength 630 nm) 28.

### Soft agar assay

Soft agar colony formation assay was performed by suspending 5 × 10³ tumor cells in a mixture of complete medium and 0.05% Noble agar (Thermo Fisher Scientific). This cell suspension was then plated on top of a solidified 0.1% agar base layer in 6-well plates. After incubation for 21 days, colonies were stained with 0.5 mg/mL MTT (Sigma) at 37 °C for 4 h. Viable colonies (> 50 μm diameter) were quantified under a microscope 16, 18-21.

### Cellular invasion assay

For the invasion assay, tumor cells (1 × 10⁵) in 200 μL serum-free medium were plated in the upper chamber of Matrigel-coated (1 : 8 dilution) Transwell inserts. The lower chamber was filled with 600 μL of medium supplemented with 10% FBS. Following a 24-h incubation at 37 °C with 5% CO₂, non-invading cells were removed from the upper surface. Cells on the lower membrane surface were then fixed with 4% paraformaldehyde for 15 min at room temperature and stained with 0.1% crystal violet for 20 min. After excision, the membranes were mounted on microscope slides for brightfield imaging, and invaded cells were quantified using established criteria 16, 18-21.

### Animal model studies

All animal procedures were approved by the Experimental Animal Ethics Committee of Huazhong University of Science and Technology (Approval No. 2019-3184) and conducted in compliance with its guidelines. For tumorigenesis and experimental metastasis assays, BALB/c nude mice (*n* = 5 per group) were randomly allocated and subjective to subcutaneous inoculation of tumor cells (1 × 10⁶) into dorsal flank or intravenous injection of tumor cells (1 × 10⁶) via the tail vein 16, 18-21. To deplete gastrointestinal fungi, NSG mice (*n* = 5 per group) received oral gavage of 200 μg amphotericin B daily for five consecutive days, followed by continuous exposure to 0.5 μg/mL amphotericin B in drinking water for 20 days. After completion of anti-fungal therapy,* M. globosa* (1 × 10^8^ CFU/mL) was administered to repopulate specie-specific fungi by oral gavage, while control group was given PBS by gavage. After 7 days of fungal administration, NSG mice (*n* = 5 per group) were randomly allocated to receive tumor cell injection (1 × 10⁶ cells in 100 μL PBS) into renal capsule 29. One week later, 60 mg/kg TCZ or PBS were injected intraperitoneally every other day. After a period of one month, mice were euthanized to assess tumor mass. For* in vivo* evaluation, tumor cells (1 × 10⁶) were injected either subcutaneously into the dorsal flank or intravenously via the tail vein into BALB/c nude mice; each experimental group consisted of five animals. One week post-inoculation, mice were randomly allocated to receive intravenous administration of MalaEx and intraperitoneal injection of TCZ or UU-T02 (60 mg/kg/day) 16, 18-21. Tumor volumes and survival of mice were recorded. *In vivo* imaging was conducted weekly with VIS^®^ Lumina III (PerkinElmer, Waltham, MA).

### Patient specimens

This study protocol was reviewed and approved by the Institutional Review Board of Union Hospital, Tongji Medical College (Approval No. 2023-0519), and was conducted in compliance with both committee's ethical guidelines and principles of the Declaration of Helsinki. Informed written consent was secured from the legal guardians of all participating children. NB tissues were obtained from surgical procedures at Union Hospital, excluding patients with prior chemotherapy or radiotherapy. Tissue samples were immediately snap-frozen in liquid nitrogen. Following this, their histological identity was verified by a board-certified pathologist. The confirmed specimens were then transferred to a -80 °C freezer for long-term storage until subsequent analysis.

### Immunohistochemistry

For immunohistochemistry, standard protocols 16, 18-21 were employed. Primary antibodies included anti-Ki-67 (1:100; ab92742), anti-CD31 (1:200; ab28364, Abcam), and mAb414 (1:200; ab24609, Abcam). Specificity controls comprised antigen-blocking peptides and isotype-matched IgG. Two blinded pathologists independently scored the immunoreactivity intensity.

### Statistical analysis

All quantitative data are summarized as mean ± standard error of the mean (SEM). For gene expression, thresholds were set based on average values; association between gene sets was tested with Fisher's exact test, and correlation was quantified using Pearson's coefficient. Differences between groups were analyzed with the Mann-Whitney U test (non-parametric data), one-way ANOVA (multiple groups), or Student's *t*-test (parametric data). Patient survival was plotted via Kaplan-Meier curves, and differences were assessed with the log-rank test. Statistical significance was determined based on two-tailed *P* values, with a threshold of FDR-adjusted *P* < 0.05.

## Results

### ARMC12 interacts with transcription factor MYC in liquid condensates

**Figure 1:**
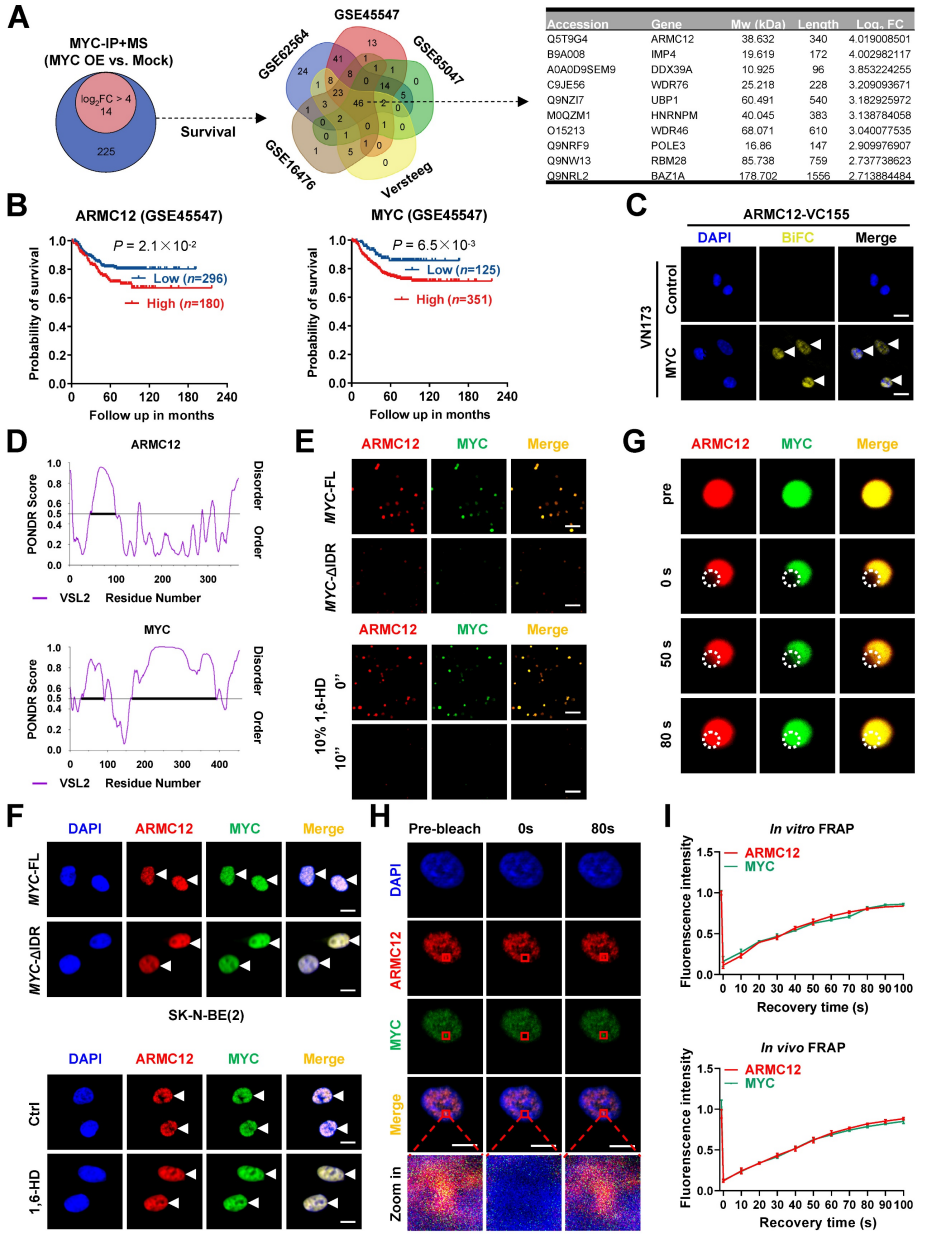
** ARMC12 interacts with transcription factor MYC within liquid condensates in NB cells.** (**A**) Venn diagrams (left and middle panels) showing the identification of differential protein partners [fold change (FC) > 2] of MYC by co-IP coupled with mass spectrometry in SK-N-BE(2) cells stably transfected with empty vector (mock) or *MYC*, and those consistently associated with survival of 498 (GSE62564), 649 (GSE45547), 283 (GSE85047), 122 (Versteeg), and 88 (GSE16476) NB patients. Table (right panel) revealing top 10 protein partners ranking by FC. (**B**) Kaplan-Meier curve showing overall survival of 649 NB patients (GSE45547) with high or low expression of *ARMC12* or *MYC* (cutoff values = 7.63 and 12.00). (**C**) Confocal images of BiFC assay indicating direct interaction between ARMC12 and MYC (arrowheads) in SK-N-BE(2) cells co-transfected with ARMC12-VC155 and MYC-VN173 constructs. Scale bars: 10 μm. (**D**) IDR within ARMC12 and MYC proteins analyzed by PONDR (http://www.pondr.com/) program. (**E**) Representative images indicating the liquid droplet formation of ARMC12-mCherry and wild-type or IDR-deficient MYC-EGFP *in vitro*, and those incubated with vehicle or 10% 1,6-HD. Scale bars: 10 um. (**F**) Fluorescence imaging assay indicating the condensate formation (arrowheads) of ARMC12 and MYC-EGFP in SK-N-BE(2) cells stably transfected with full-length or IDR-deficient* MYC* construct, and those incubated with vehicle or 10% 1,6-HD. Scale bars: 10 μm. (**G**) Representative images of FRAP assay showing the exchange kinetics (circles) of ARMC12-mCherry and MYC-EGFP within condensates. Scale bars: 10 μm. (**H**) Representative images of FRAP assay showing the exchange kinetics (boxes) of ARMC12 and MYC-EGFP in SK-N-BE(2) cells stably transfected with *MYC* construct. Scale bars: 10 μm. (**I**) Quantification of FRAP assay indicating the exchange kinetics of ARMC12-mCherry and MYC-EGFP within condensates *in vitro*, and that of ARMC12 and MYC-EGFP *in vivo*. Fisher's exact test for over-lapping analysis in **A**. Log-rank test for survival comparison in **B**. Data are shown as mean ± s.e.m. (error bars) or representative of three independent experiments in **C** and** E-I**.

Our previous studies have indicated the various MYC expression in cultured NB cell models 16. For comprehensively profiling the MYC interactome, co-IP coupled with mass spectrometry was performed in SK-N-BE(2) cells (representing relatively low *MYC* levels) receiving transfection of empty vector or *MYC* construct. The results revealed 239 candidate proteins enriched in *MYC* over-expression group (fold changer > 2, *P* < 0.01, Figure 1A, Figure S1A and Table S4). Comprehensive analysis indicated that 46 proteins were consistently correlated with patients' survival across five independent NB cohorts (*n* = 498, 649, 283, 122, and 88 from datasets GSE62564, GSE45547, GSE85047, Versteeg, and GSE16476, respectively; Figure 1A). Among them, ARMC12 was top candidate ranking by enrichment fold (Figure 1A and Figure S1B). Elevated *ARMC12* or *MYC* expression was linked to an adverse prognosis in NB cohorts (Figure 1B). Endogenous co-localization of ARMC12 and MYC protein was observed in ganglioneuroblastoma (GNB) or NB specimens, while their Pearson's coefficient was higher in NB tissues (Figure S1C). In addition, ARMC12 was co-localized with MYC in the cellular context of SH-SY5Y and SK-N-AS (Figure S1D). Endogenous ARMC12-MYC complex formation was further confirmed in these NB cells exhibiting intermediate *MYC* expression (Figure S1E). BiFC assay indicated the complementary fluorescence in SK-N-BE(2) cells co-transfected by ARMC12-VC155 and MYC-NV173 constructs (Figure 1C). Domain mapping experiments indicated that 1-150 amino acids (aa) of MYC was necessary for ARMC12 binding (Figure S1F). Meanwhile, armadillo 2 (ARM2, 206-245 aa) domain, but not armadillo 1 (ARM1, 127-205 aa) or armadillo 3 (ARM3, 246-345 aa) domain, of ARMC12 protein mediated its binding to MYC in SK-N-BE(2) cells harboring FLAG-tagged *ARMC12* and HA-tagged* MYC* constructs (Figure S1F). Given the presence of substantial intrinsically disordered regions (IDRs) in both ARMC12 and MYC, as predicted by the PONDR program 30 (Figure 1D), we next sought to investigate their potential to undergo liquid-liquid phase separation (LLPS). Imaging with high-resolution fluorescence microscopy revealed that purified recombinant ARMC12-mCherry and MYC-EGFP proteins (purity > 90%; Figure S2A) underwent spontaneous condensation to form biomolecular condensates *in vitro* (Figure 1E). Meanwhile, deletion of the IDR in MYC abolished the ability of recombinant MYC-EGFP and ARMC12-mCherry proteins to undergo phase separation *in vitro* (Figure 1E). Consistent with this, pharmacological disruption of LLPS using 1,6-hexanediol (1,6-HD) 31 caused the dissociation of ARMC12-mCherry and MYC-EGFP condensates *in vitro* (Figure 1E). To examine the physiological relevance, we performed fluorescence imaging in SK-N-BE(2) cells, which revealed analogous compartmentalization of ARMC12 and MYC-EGFP *in vivo* (Figure 1F). Transfection of MYC-EGFP construct with IDR deletion or treatment with 1,6-HD reduced the LLPS of ARMC12 and MYC-EGFP proteins in NB cells (Figure 1F). To assess the dynamic mobility of these condensates, FRAP assays were performed. Both *in vitro* and *in vivo*, ARMC12 and MYC within the droplets exhibited rapid fluorescence recovery (Figure 1G-I), indicating their liquid-like molecular mobility. These findings demonstrated that ARMC12 physically interacted with MYC within liquid biomolecular condensates.

### ARMC12/MYC interplay in liquid condensates to drive NPC biogenesis

**Figure 2:**
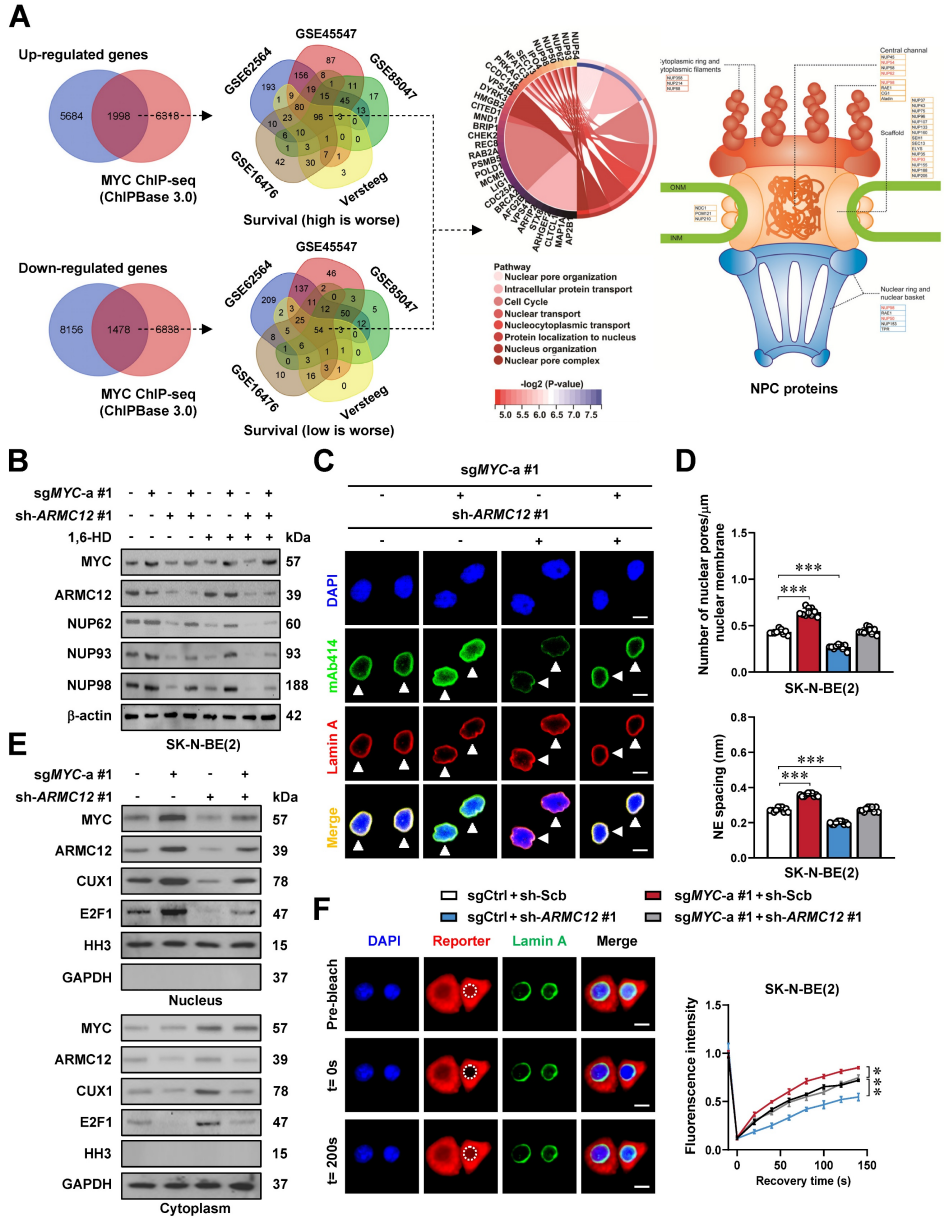
** ARMC12/MYC interplay in liquid condensates to drive NPC biogenesis.** (**A**) Venn diagrams of RNA-seq assay (left and middle panels) indicating the alteration of gene expression (fold change > 1.5, *P* < 0.05) in SH-SY5Y cells stably transfected with empty vector (mock) or *ARMC12*, and those consistently associated with survival of 498 (GSE62564), 649 (GSE45547), 283 (GSE85047), 122 (Versteeg), and 88 (GSE16476) NB patients. The chord and illustration diagrams (middle and right panels) displaying the involvement of identified 150 genes in GO pathways and NPC formation. (**B**) Western blot assay showing the expression of MYC, ARMC12, NUP62, NUP93, and NUP98 in SK-N-BE(2) cells stably transfected with control sgRNA (sgCtrl), sg*MYC*-a #1, scramble shRNA (sh-Scb), or sh-*ARMC12* #1, and those treated with vehicle or 10% 1,6-HD. (**C**) Representative images of immunofluorescence assay indicating the localization and number of NPC (arrowheads) in SK-N-BE(2) cells stably transfected with sgCtrl, sg*MYC*-a #1, sh-Scb, or sh-*ARMC12* #1. (**D**) Quantification of transmission electron microscopy indicating the NPC numbers and nuclear envelope (NE) spacing in SK-N-BE(2) cells stably transfected with sgCtrl, sg*MYC*-a #1, sh-Scb, or sh-*ARMC12* #1. (**E**) Western blot assay showing the nuclear or cytoplasmic distribution of MYC, ARMC12, CUX1, or E2F1 in SK-N-BE(2) cells stably transfected with sgCtrl, sg*MYC*-a #1, sh-Scb, or sh-*ARMC12* #1. (**F**) Representative images (left panel) and quantification (right panel) of FRAP assay indicating the exchange kinetics (circles) of NLS-mCherry-NES reporter within SK-N-BE(2) cells stably transfected with sgCtrl, sg*MYC*-a #1, sh-Scb, or sh-*ARMC12* #1. One-way ANOVA compared the difference in **D** and **F**. * *P* < 0.05, ** *P* < 0.01, *** *P* < 0.001. Data are shown as mean ± s.e.m. (error bars) or representative of three independent experiments in **B-F**.

To further identify downstream targets regulated by ARMC12/MYC in SH-SY5Y cells, RNA-seq transcriptomic analysis was performed following *ARMC12* over-expression. This approach revealed 7682 up-regulated and 9634 down-regulated transcripts [fold change (FC) > 1.5, false discovery rate (FDR)-adjusted *P* < 0.05] (Figure 2A). Over-lapping analysis with ChIP-seq datasets in ChIPBase 3.0 32 revealed that 1998 up-regulated and 1478 down-regulated genes were MYC downstream targets, while 96 and 54 of them were consistently associated with unfavorable or favorable outcome of 498 (GSE62564), 649 (GSE45547), 283(GSE85047), 122 (Versteeg), and 88 (GSE16476) NB patients, respectively (Figure 2A and Table S5). These genes were mainly involved in nuclear pore organization or nuclear transport, including essential NPC components *NUP50*, *NUP54*, *NUP62*, *NUP93*, and *NUP98* (Figure 2A). Mining of a public ChIP-seq dataset (GSE138295) confirmed the enrichment of MYC on promoter regions of *NUP50*, *NUP54*, *NUP62*, *NUP93*, and *NUP98* in NB cells (Figure S2B). Meanwhile, knockdown of *MYC* in SH-SY5Y cells reduced its binding and transcript levels of *NUP62*, *NUP93*, and *NUP98*, but not of *NUP50* or *NUP54* (Figure S2C). Following *MYC* silencing, the promoter activity of *NUP62*, *NUP93*, and *NUP98* was decreased in a MYC binding site-dependent manner (Figure S2C). Genomic analysis of 2001 NB cases from cBioportal database (https://www.cbioportal.org/) revealed no detectable mutations (missense/nonsense, insertions, or deletions) in *NUP62*, *NUP93*, or *NUP98* (Figure S2D). Instead, higher levels of *NUP62*, *NUP93*, and *NUP98* were correlated with mortality, high-risk, advanced stages of International Neuroblastoma Staging System (INSS), or disease progression in 498 NB patients (GSE62564, Figure S2E and Figure S3), and associated with worse survival outcome (Figure S4A-C). In SK-N-BE(2) and SH-SY5Y cells with *MYC* activation or suppression by clustered regularly interspaced short palindromic repeats (CRISP)-deactivated Cas9 (dCas9) 33, dual-luciferase reporter, ChIP-qPCR, real-time qRT-CPR, and immunoblotting analyses indicated the increase or decrease of MYC activity, along with elevation or reduction in MYC enrichment, promoter activity, and expression of target genes (*NUP62*, *NUP93*, and *NUP98*), which was abolished by *ARMC12* knockdown or over-expression, respectively (Figure 2B and Figure S5A-F). The interplay of ARMC12 and MYC in regulating expression of these genes was attenuated by 1,6-HD treatment (Figure 2B and Figure S5A-F). Importantly, following induced over-expression of *MYC* in SK-N-BE(2) cells, there was a significant increase in the NPC number and nuclear envelope (NE) spacing, which was attenuated by *ARMC12* silencing (Figure 2C, D and Figure S6A). Additionally, the number of NPC was increased in NB tissues, when compared to that of GNB specimens (Figure S6B).

Since gene set enrichment analysis (GSEA) indicated the target genes of transcriptional regulators, such as MYC, CUX1, and E2F1, in *AMRC12* over-expressing NB cells (Figure S6C), we further observed the potential impact of *ARMC12* and *MYC* on their nuclear translocation. Subcellular fraction and immunoblotting analyses revealed the elevation or reduction in the nuclear abundance of transcriptional regulators MYC, ARMC12, CUX1, and E2F1 upon *MYC* activation or suppression (Figure 2E and Figure S6D). In FRAP assay using an NLS-mCherry-NES reporter, confocal observation also demonstrated the increase of nuclear transport in SK-N-BE(2) cells with constitutive *MYC* over-expression (Figure 2F). Meanwhile, *ARMC12* depletion or over-expression reversed the alteration of nuclear transport induced by *MYC* modulation (Figure 2E, F and Figure S6D). Notably, elevated *ARMC12* and *MYC* levels were also linked to poor outcome of adrenocortical sarcinoma, B-cell lymphoma, renal clear cell carcinoma, or breast tumor (Figure S7A-D). These results indicated that ARMC12/MYC interplay in liquid condensates drove NPC biogenesis.

### *ARMC12*/*MYC* interplay promotes tumorigenesis and aggressiveness of NB

**Figure 3:**
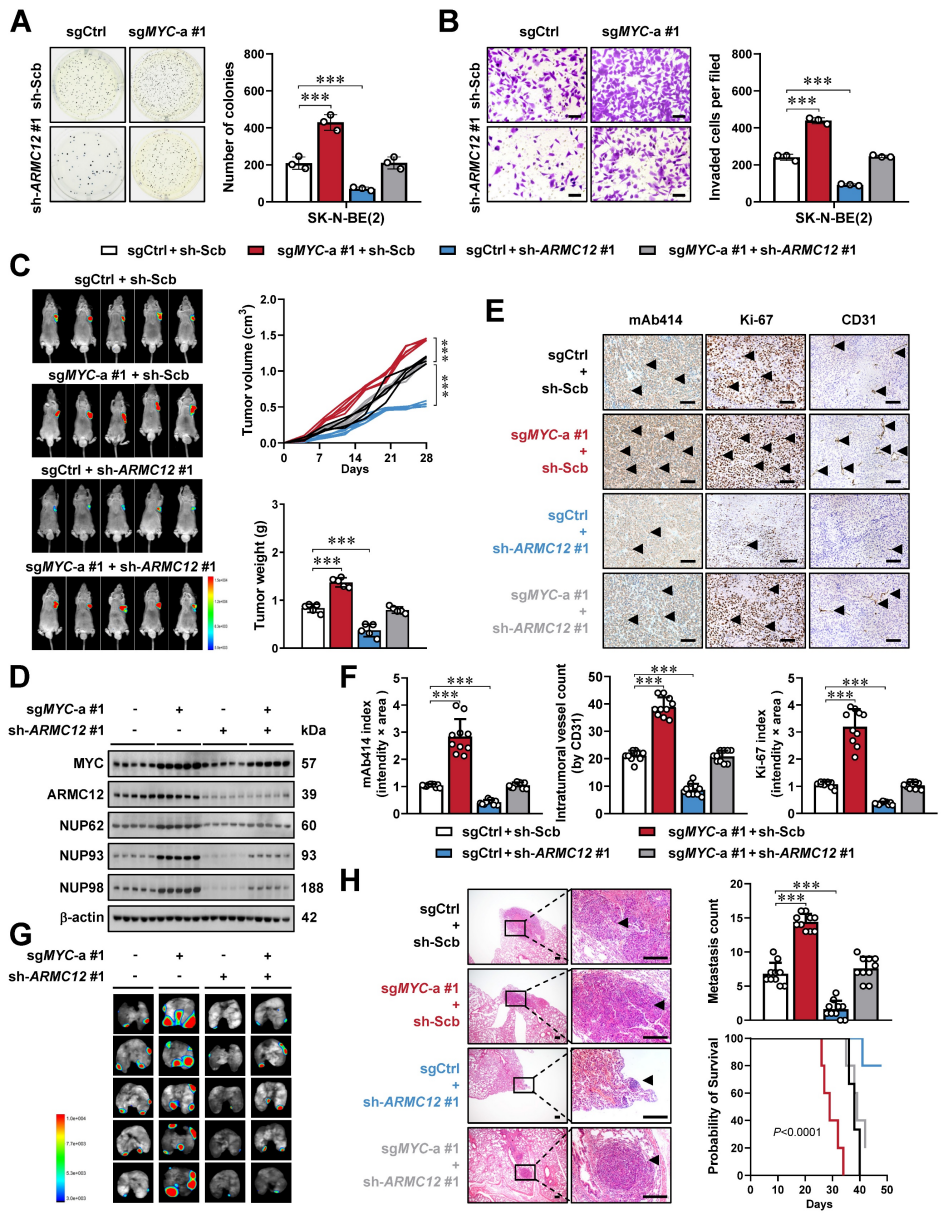
***ARMC12*/*MYC* interplay promotes tumorigenesis and aggressiveness of NB *in vitro* and *in vivo*.** (**A** and **B**) Representative images (left panel) and quantification (right panel) of soft agar (A) and Matrigel invasion (B) assays showing the growth and invasion of SK-N-BE(2) cells stably transfected with control sgRNA (sgCtrl), sg*MYC*-a #1, scramble shRNA (sh-Scb), or sh-*ARMC12* #1 (*n* = 3). Scale bars: 50 μm. (**C**) Representative images (left panel), growth curve (right upper panel), and weight (right lower panel) of subcutaneous xenograft tumors formed by SK-N-BE(2) cells stably transfected with sgCtrl, sg*MYC*-a #1, sh-Scb, or sh-*ARMC12* #1 in nude mice (*n* = 5 per group). (**D**) Western blot assay showing the expression of MYC, ARMC12, NUP62, NUP93, and NUP98 in xenograft tumors formed by SK-N-BE(2) cells stably transfected with sgCtrl, sg*MYC*-a #1, sh-Scb, or sh-*ARMC12* #1 in nude mice. (**E** and** F**) Representative images (E) and quantification (F) of immunohistochemistry indicating the expression of NPC, Ki-67, and CD31 (arrowheads) in xenograft tumors formed by SK-N-BE(2) cells stably transfected with sgCtrl, sg*MYC*-a #1, sh-Scb, or sh-*ARMC12* #1 in nude mice (*n* = 5 per group). Scale bars: 100 μm. (**G** and **H**) Representative images (G), H&E staining (H, arrowheads) or quantification (H) of lung metastasis, and Kaplan-Meier curves (H) of nude mice treated with tail vein injection of SK-N-BE(2) cells stably transfected with sgCtrl, sg*MYC*-a #1, sh-Scb, or sh-*ARMC12* #1 in nude mice (*n* = 5 per group). Scale bars: 150 μm. One-way ANOVA compared the difference in **A**-**C**, **F** and **H**. Log-rank test for survival comparison in **H**. *** *P* < 0.001. Data are shown as mean ± s.e.m. (error bars) or representative of three independent experiments in **A-H**.

To determine whether ARMC12 functionally intersects with MYC to drive NB aggressiveness, a genetic rescue strategy was employed. Compensatory modulation of *ARMC12* expression rescued the oncogenic phenotypes resulting from stable *MYC* activation or suppression in SK-N-BE(2) and SH-SY5Y cell lines, normalizing their growth and invasive properties (Figure 3A, B and Figure S7E, F). Stable *ARMC12* depletion rescued the *MYC*-driven tumor-promoting phenotype in SK-N-BE(2) xenografts in nude mice. Specifically, it reversed enhancement in tumor growth, mass, and expression of downstream genes (*NUP62*, *NUP93*, and *NUP98*) (Figure 3C, D), accompanied by modulation of NPC numbers and Ki-67 or CD31 labeling index (Figure 3E, F). In addition, tail vein injection of *MYC* over-expressing SK-N-BE(2) cells into nude mice led to a marked increase in lung metastasis and a concomitant decline in overall condition, as evidenced by shortened survival and decreased body weight, and these alterations were reversed by *ARMC12* knockdown (Figure 3G, H and Figure S7G). Above findings indicated that *ARMC12*/*MYC* interplay promoted tumorigenesis and aggressiveness of NB.

### Tioconazole inhibits the ARMC12-MYC interaction in NB cells

**Figure 4:**
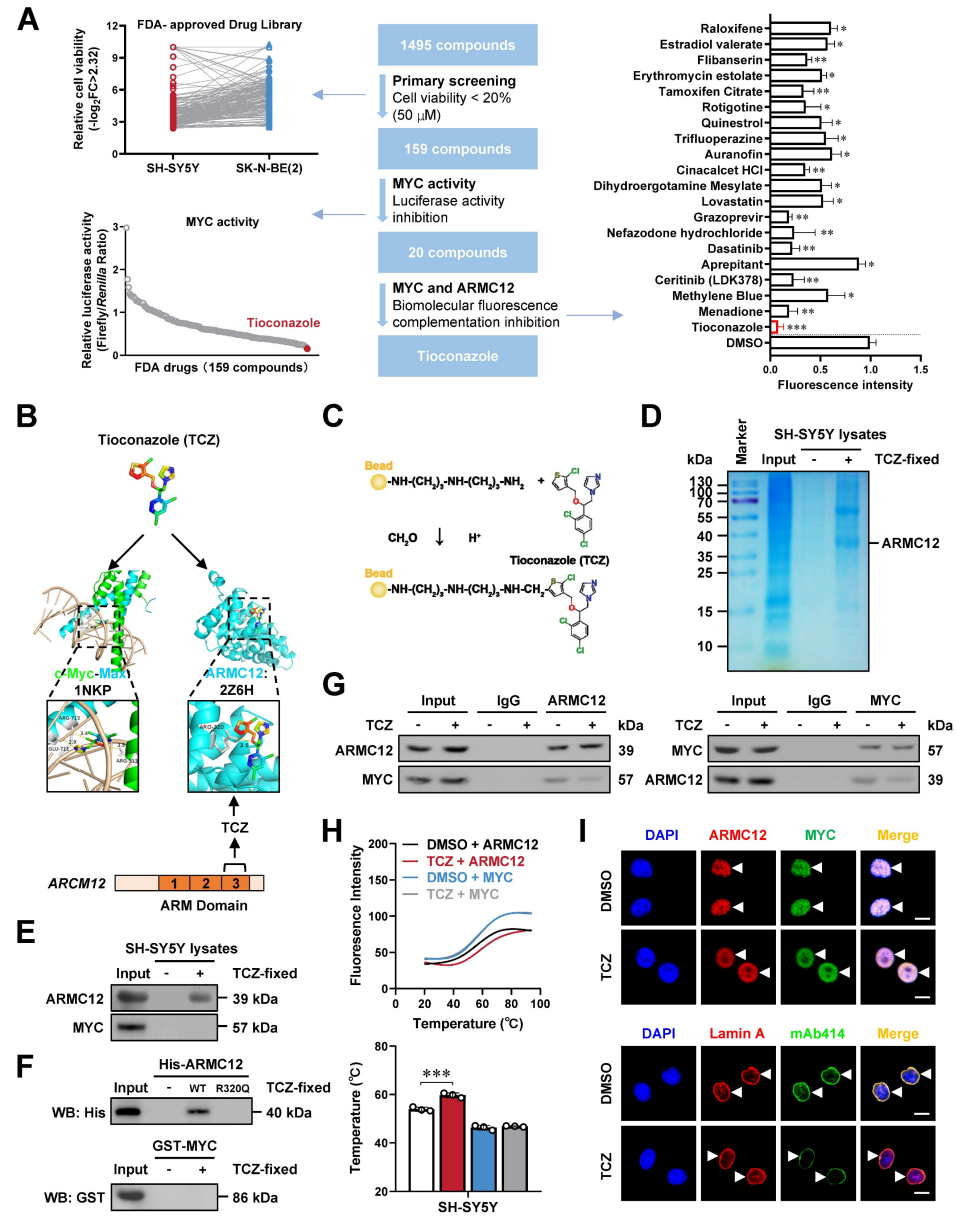
** Tioconazole inhibits the interaction between ARMC12 and MYC in NB cells.** (**A**) MTT colorimetric, dual-luciferase, and BiFC assays revealing the screening of ARMC12-MYC interaction inhibitor from 1495 FDA-approved drugs in NB cells. (**B**) Molecular docking analysis of TCZ with MYC or ARMC12 protein via HDOCK program (http://hdock.phys.hust.edu.cn/). (**C**-**E**) Chemical structure (C), Coomassie blue staining (D), and western blot (E) assays showing the binding of ARMC12 or MYC within SH-SY5Y cell lystaes to NHS beads covalently conjugated with TCZ (20 μM). (**F**) Western blot assay indicating the affinity of recombinant wild-type or R320Q mutant ARMC12 or MYC protein to NHS beads covalently conjugated with TCZ (20 μM). (**G**) Co-IP and western blot assays showing the interaction between ARMC12 and MYC in SH-SY5Y cells treated with solvent or TCZ (20 μM). (**H**) DSF assay (upper panel) and temperature (lower panel) showing the fluroresence intensity of His-tagged ARMC12 or GST-tagged MYC protein incubated with DMSO or TCZ (20 μM). (**I**) Fluorescence imaging assay (upper panel) indicating the condensate formation (arrowheads) of ARMC12 and MYC-EGFP in SK-N-BE(2) cells stably transfected with *MYC* construct, and those incubated with DMSO or TCZ (20 μM). Representative images (lower panel) of immunofluorescence assay indicating the localization and number of NPC (arrowheads) in SK-N-BE(2) cells incubated with DMSO or TCZ (20 μM). Scale bars: 10 μm. One-way ANOVA compared the difference in **A** and **H**. * *P* < 0.05, ** *P* < 0.01, *** *P* < 0.001. Data are shown as mean ± s.e.m. (error bars) or representative of three independent experiments in **A** and **D**-**I**.

To identify inhibitors of AMRC12-MYC interaction, NB cells were exposed to 1495 Food and Drug Administration (FDA)-approved drugs (Figure 4A). Among them, 159 compounds reduced cell viability by over 80% (Figure 4A, Table S6). Subsequent dual-luciferase assay revealed that 20 of these compounds suppressed MYC activity by more than 80% (Figure 4A, Table S6). BiFC screening identified tioconazole (TCZ), menadione, and ceritinib as inhibitors of ARMC12-MYC interaction, with TCZ showing the strongest suppression (Figure S8A). In cultured NB cells, TCZ inhibited the viability in a dose-dependent manner (Figure S8B). The HDOCK molecular docking program (http:// hdock.phys.hust.edu.cn/) predicted the binding of TCZ to ARM3 domain, especially amino acid residue 320 (3.5 Å distance), of ARMC12 protein (Figure 4B). Affinity purification 26 and mass spectrometry assays indicated 146 TCZ-enriched proteins from SH-SY5Y cell lystaes (Table S7), while 16 candidates were linked to survival outcomes in five publicly available NB datasets, encompassing 498 (GSE62564), 649 (GSE45547), 283 (GSE85047), 122 (Versteeg), and 88 (GSE16476) patients (Figure S9A). Among them, ARMC12 was top candidate ranking by enrichment fold (Figure S9A). Validating affinity purification and western blot assays indicated the TCZ-enriched ARMC12, but not MYC protein, from SH-SY5Y cell lystaes (Figure 4C-E). Moreover, TCZ directly interacted with recombinant wild-type ARMC12 protein rather than its mutant in amino acid residue 320 or recombinant MYC protein (Figure 4F). Importantly, following TCZ treatment, the interaction between ARMC12 and MYC was markedly weakened in SH-SY5Y and SK-N-AS cells, as evidenced by co-IP coupled with western blot analyses (Figure 4G and Figure S9B), and this inhibitory effect was biochemically confirmed through DSF measurements 27 (Figure 4H). Meanwhile, mutation of 320^th^ amino acid reversed the repressive effects of TCZ on ARMC12-MYC interaction (Figure S9C), and prevented the decrease in MYC activity, MYC enrichment, promoter activity, and expression of target genes (*NUP62*, *NUP93*, *NUP98*) induced by TCZ treatment in SH-SY5Y cells (Figure S9D-F). Of note, TCZ treatment reduced the LLPS of ARMC12 and MYC (Figure 4I), and decreased the NPC number in SH-SY5Y cells (Figure 4I). These results suggested that TCZ inhibited the ARMC12-MYC interaction in NB cells.

### *M. globosa* extracellular vesicles facilitate NPC biogenesis and tumorigenesis

**Figure 5:**
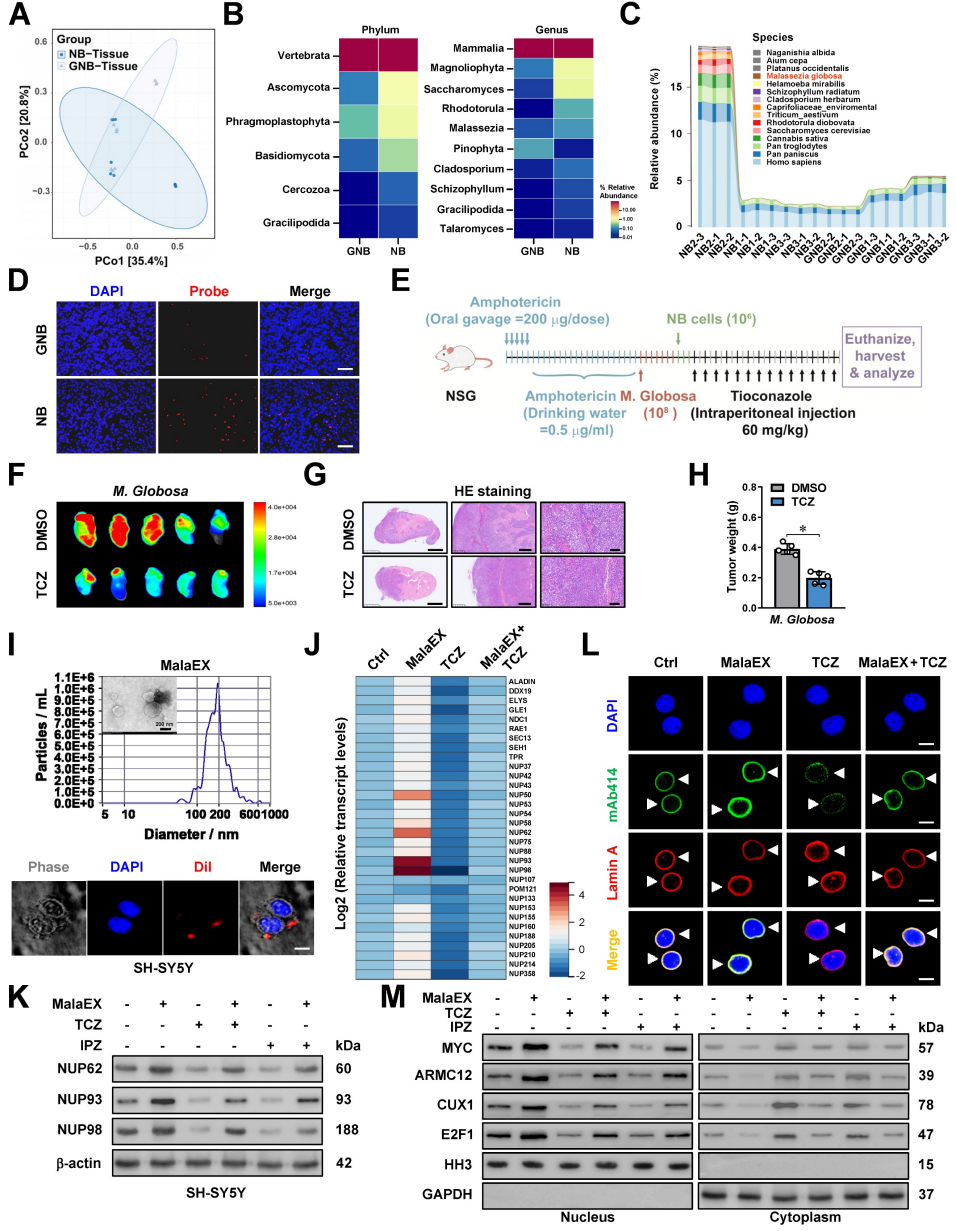
***M. globosa* extracellular vesicles facilitate NPC biogenesis and tumorigenesis.** (**A-C**) Mycobiome analysis using 18S rRNA sequencing indicating the principal components (PC, A), phylum (B), genus (B), and species (C) of fungi within NB and GNB tissues. (**D**) Fungal FISH assay validating the elevated abundance of *M. globosa* in NB tissues, than that of GNB specimens. (**E**-**G**) Representative images (F) and H&E staining (G) showing the growth and renal invasion of SH-SY5Y-formed xenograft tumors under renal capsule of NSG mice receiving oral gavage of amphotericin B, *M. globosa*, and intraperitoneal injection of TCZ as indicated (E). (**H**) Tumor weight of NSG mice receiving oral gavage of amphotericin B, *M. globosa*, and intraperitoneal injection of TCZ. (**I**) Electron microscopic analysis and particle size assay (upper panel) validating the EVs extracted from culture medium of *M. globosa* (MalaEX), with the uptake of Dil-labeled EVs by NB cells observed under a confocal microscope. (**J**) Real-time qRT-PCR assay revealing the transcript levels (normalized to *β-actin*, *n* = 5) of NPC biogenesis genes in SH-SY5Y cells treated with MalaEx or TCZ (20 μM). (**K**) Western blot assay showing the expression of NUP62, NUP93, and NUP98 in SH-SY5Y cells treated with MalaEx, TCZ (20 μM), or IPZ (20 μM). (**L**) Representative images of immunofluorescence assay indicating the localization and number of NPC (arrowheads) in SH-SY5Y cells treated with MalaEx or TCZ (20 μM). (**M**) Western blot assay showing the nuclear or cytoplasmic distribution of MYC, ARMC12, CUX1, or E2F1 in SH-SY5Y cells treated with MalaEx, TCZ (20 μM), or IPZ (20 μM). Student's *t* test compared the difference in **H**. * *P* < 0.05. Data are shown as mean ± s.e.m. (error bars) or representative of three independent experiments in **C**, **D** and **F**-**M**.

Since TCZ is an established anti-fungal drug, we hypothesized that it might also affect the intratumoral fungi driving tumorigenesis. The mycobiome analysis using 18S rRNA sequencing indicated the consistent existence of fungi within NB and GNB tissues (Figure 5A). Of note, fungi of various phylum, class, order, family, or genus were differentially detected between NB and GNB specimens (Figure 5B, Figure S10A-C and Table S8). Among them, *M. globosa*, an oncogenic fungi driving PDAC progression 24, was obviously enriched in NB tissues (Figure 5C) and chosen for further studies. Fungal FISH validated the elevated abundance of *M. globosa* in NB tissues (Figure 5D). To evaluate the effects of *M. globosa* on NB progression, amphotericin B pretreatment was performed in NSG mice prior to oral gavage administration of fungal strain (Figure 5E). There was significant *M. globosa* colonization, growth, and renal invasion of SH-SY5Y-derived xenograft tumors under renal capsule of these NSG mice, which was attenuated by intraperitoneal injection of TCZ (Figure 5E-G and Figure S10D, E), accompanying by decrease in tumor weight (Figure 5H). Since previous studies revealing the oncogenic roles of fungi EVs 34, we isolated EVs from *M. globosa* culture medium (designated MalaEX) and confirmed their structural integrity through electron microscopy and particle size analysis (Figure 5I). The internalization efficiency of Dil-labeled EVs by NB cells was subsequently quantified via immunofluorescence assay (Figure 5I). MalaEX incubation up-regulated the mRNA expression of *NUP62*, *NUP93*, and *NUP98* in SH-SY5Y cells, whereas TCZ treatment reversed this effect (Figure 5J). Western blot assay revealed that MalaEX treatment up-regulated NUP62, NUP93, and NUP98 protein levels of NB cells (Figure 5K and Figure S10F). However, this upregulation was abolished upon administration of TCZ or importazole (IPZ), a validated inhibitor targeting importin-β-dependent nuclear transport pathway (Figure 5K and Figure S10F). Consistently, NPC biogenesis of SH-SY5Y cells was facilitated by MalaEX incubation, while TCZ treatment attenuated these effects (Figure 5L). The nuclear translocation of MYC, ARMC12, and other transcriptional regulators (CUX1 or E2F1) was enhanced in NB cells incubated with MalaEX, which was reversed by TCZ or IPZ treatment (Figure 5M and Figure S10G). These results suggested that *M. globosa* extracellular vesicles facilitated NPC biogenesis and tumorigenesis.

### MGL_0381 facilitates MYC transactivation and NPC biogenesis

**Figure 6:**
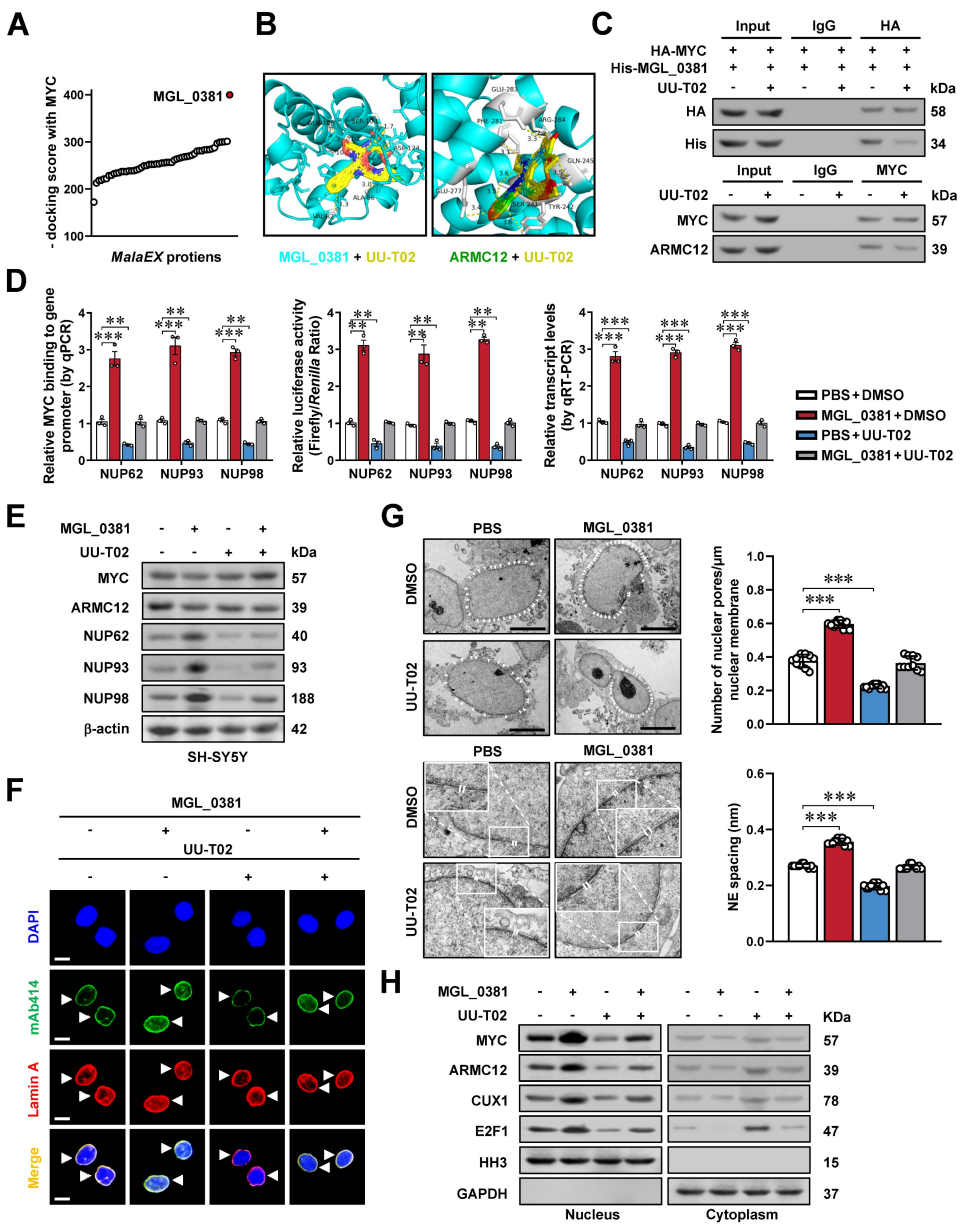
** MGL_0381 facilitates MYC transactivation and NPC biogenesis.** (**A**) Molecular docking of MalaEX content proteins with MYC via HDOCK program (http://hdock.phys.hust.edu.cn/). (**B**) Molecular docking of MGL_0381 or ARMC12 protein with UU-T02 via HDOCK program. (**C**) Co-IP and western blot assays showing the interaction between HA-tagged MYC and His-tagged MGL_0381 protein in SK-N-BE(2) cells transfected with *MYC* construct, endogenous interaction of MYC with ARMC12 in SH-SY5Y cells, and those incubated with DMSO or UU-T02 (1.0 μM). (**D**) ChIP-qPCR (normalized to input, *n* = 3), dual-luciferase (*n* = 3), and real-time qRT-PCR (normalized to *β-actin*, *n* = 3) assays indicating the MYC enrichment, promoter activity, and transcript levels of *NUP62*, *NUP93*, or *NUP98* in SH-SY5Y cells treated with PBS or recombinant MGL_0381, and those incubated with DMSO or UU-T02 (1.0 μM). (**E**) Western blot assay showing the expression of MYC, ARMC12, NUP62, NUP93, and NUP98 in SH-SY5Y cells treated with PBS or recombinant MGL_0381, and those incubated with DMSO or UU-T02 (1.0 μM). (**F** and **G**) Representative images and quantification of immunofluorescence assay (F) and transmission electron microscopy (G) indicating the NPC number (arrowheads) and nuclear envelope (NE) spacing in SH-SY5Y cells treated with PBS or recombinant MGL_0381, and those incubated with DMSO or UU-T02 (1.0 μM). (**H**) Western blot assay showing the nuclear or cytoplasmic distribution of MYC, ARMC12, CUX1, or E2F1 in SH-SY5Y cells treated with PBS or recombinant MGL_0381, and those incubated with DMSO or UU-T02 (1.0 μM). One-way ANOVA compared the difference in **D** and** G**. ** *P* < 0.01, *** *P* < 0.001. Data are shown as mean ± s.e.m. (error bars) or representative of three independent experiments in **C**-**H**.

To further investigate the roles of MalaEX in NPC biogenesis, we performed proteomic profiling to detect diverse protein contents (Figure S10H), followed by molecular docking analysis with MYC protein via HDOCK server (http://hdock.phys.hust.edu.cn/). Among them, MGL_0381 was found to achieve highest docking score (Figure 6A). Of note, both MGL_0381 and AMRC12 contained Armadillo-like helical or fold similar to that of β-catenin (Figure S11A), while their potential binding with UU-T02, an established β-catenin inhibitor 35, was evaulated by molecular docking via HDOCK program (Figure 6B). The NHS beads-mediated affinity purification assay indicated direct interaction of UU-T02 with recombinant MGL_0381 or ARMC12, but not with MYC protein (Figure S11B, C and Table S9). Meanwhile, mutation of amino acid residue 242, but not of amino acid residue 241 or 245, within ARM2 domain, abolished the interaction of ARMC12 with UU-T02 (Figure S11D). In addition, UU-T02 treatment reduced the binding of MGL_0381 or ARMC12 to MYC protein in NB cells (Figure 6C). In SH-SY5Y cells, administration of recombinant MGL_0381 protein led to a significant elevation in MYC enrichment, concomitant with an augmentation in promoter activity, transcript levels, and protein expression of *NUP62*, *NUP93*, and *NUP98* (Figure 6D, E), resulting in enhanced NPC biogenesis (Figure 6F, G) and nuclear transport of transcriptional regulators (Figure 6H). Meanwhile, UU-T02 treatment reversed these alterations induced by MGL_0381 protein (Figure 6D-H). These findings suggested that as a protein content of MalaEX, MGL_0381 facilitated MYC transactivation and NPC biogenesis.

### Synergetic targeting AMRC12- and MGL_0381-facilitated MYC transactivation inhibits NPC biogenesis and NB progression

**Figure 7:**
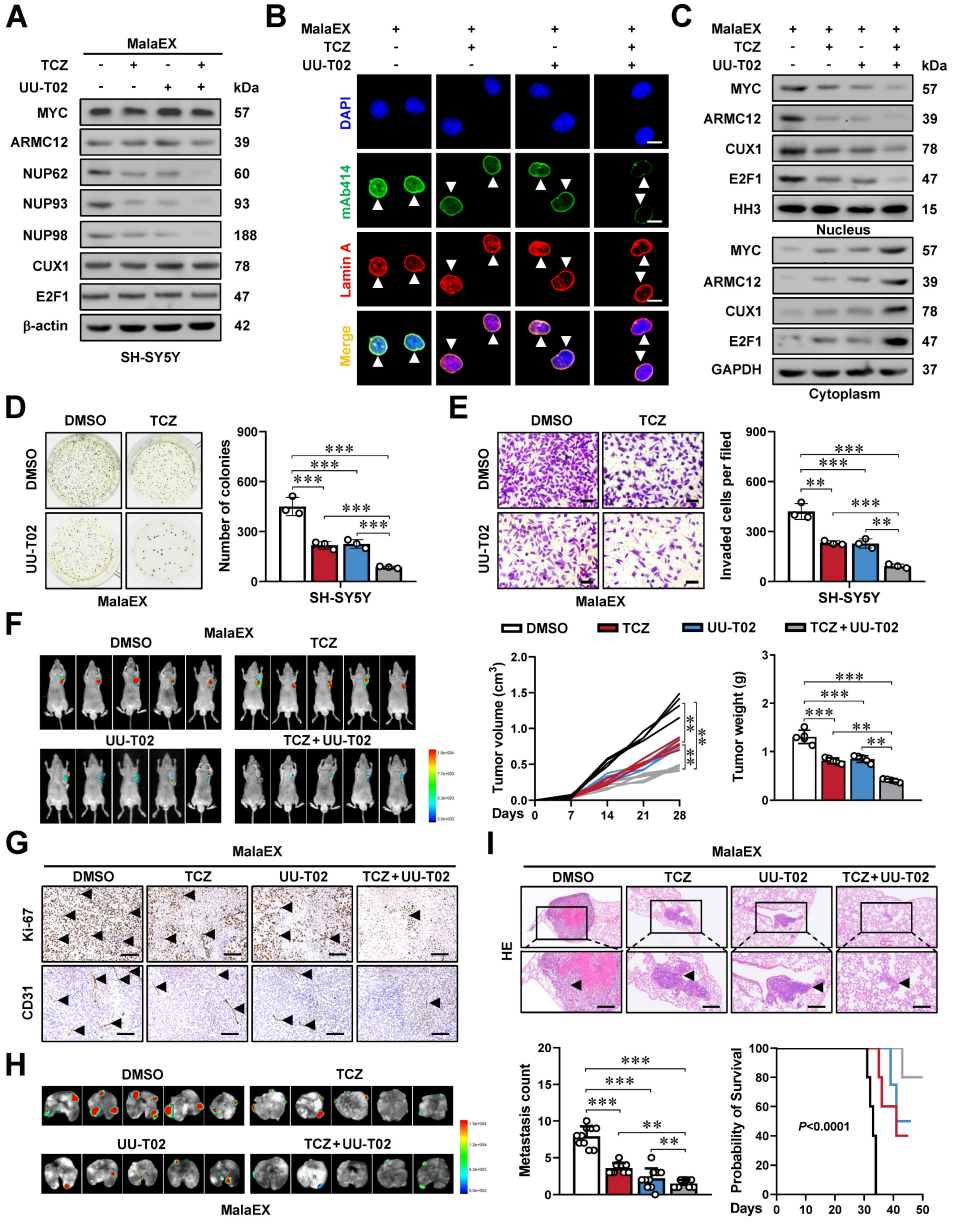
** Synergetic targeting AMRC12- and MGL_0381-facilitated MYC transactivation inhibits NPC biogenesis and NB progression.** (**A**) Western blot assay showing the expression of MYC, ARMC12, NUP62, NUP93, and NUP98 in SH-SY5Y cells treated with MalaEx, TCZ (20 μM), or UU-T02 (1.0 μM). (**B**) Representative images of immunofluorescence assay indicating the localization and number of NPC (arrowheads) in SH-SY5Y cells treated with MalaEx, TCZ (20 μM), or UU-T02 (1.0 μM). (**C**) Western blot assay showing the nuclear or cytoplasmic distribution of MYC, ARMC12, CUX1, or E2F1 in SH-SY5Y cells treated with MalaEx, TCZ (20 μM), or UU-T02 (1.0 μM). (**D** and **E**) Representative images (left panel) and quantification (right panel) of soft agar (D) and Matrigel invasion (E) assays showing the growth and invasion of SH-SY5Y cells treated with MalaEx, TCZ (20 μM), or UU-T02 (1.0 μM, *n* = 3). Scale bars: 50 μm. (**F**) Representative images (left panel), growth curve (middle panel), and tumor weight (middle panel) of subcutaneous xenograft tumors formed by SH-SY5Y cells in nude mice that received intravenous administration of MalaEx and intraperitoneal injection of TCZ (60 mg/kg/day) or UU-T02 (60 mg/kg/day, *n* = 5 per group). (**G**) Representative images of immunohistochemistry indicating the immunostaining of Ki-67 and CD31 (arrowheads) in subcutaneous xenograft tumors formed by SH-SY5Y cells in nude mice that received administration of MalaEx, TCZ (60 mg/kg/day), or UU-T02 (60 mg/kg/day). Scale bars: 100 μm. (**H** and **I**) Representative images (H), H&E staining (I, arrowheads) or quantification (I) of lung metastasis, and Kaplan-Meier curves (I) of nude mice treated with tail vein injection of SH-SY5Y cells in nude mice that received administration of MalaEx, TCZ (60 mg/kg/day), or UU-T02 (60 mg/kg/day, *n* = 5 per group). One-way ANOVA compared the difference in **D**-**F** and **I**. Log-rank test for survival comparison in **I**. ** *P* < 0.01, *** *P* < 0.001. Data are shown as mean ± s.e.m. (error bars) or representative of three independent experiments in **A-I**.

We subsequently assessed the impact of targeting AMRC12- and MGL_0381-facilitated MYC transactivation on tumorigenesis and aggressiveness. Administration of TCZ and UU-T02 directly inhibited the phase-separated biomolecular condensates of ARMC12 and MYC *in vitro* (Figure S11E), and exerted synergetic effects in reducing the MYC enrichment, promoter activation, and expression of target genes (*NUP62*, *NUP93*, *NUP98*), NPC biogenesis, and nuclear transport of transcriptional regulators in MalaEX-treated SH-SY5Y and SK-N-AS cells (Figure 7A-C, Figure S11F, and Figure S12A, B). Combined treatment with TCZ and UU-T02 synergistically suppressed both colony formation in soft agar and invasive capacity through Matrigel in MalaEX-exposed NB cells (Figure 7D, E and Figure S12C, D). The TCZ/UU-T02 combination showed more tumor suppression efficiency in SH-SY5Y xenografts compared to monotherapies, significantly inhibiting tumor volume, mass, proliferative activity (Ki-67 index), and angiogenesis (CD31^+^ vessels), with no obvious toxicity in the heart, liver, or kidney of nude mice models (Figure 7F, G and Figure S12E, F). In addition, the TCZ/UU-T02 combination administered via tail vein markedly suppressed SH-SY5Y-derived pulmonary metastasis, extended survival, and improved body weight of nude mice compared to single-agent regimens (Figure 7H, I and Figure S12G). Taken together, these results indicated that synergetic targeting AMRC12- and MGL_0381-facilitated MYC transactivation inhibited NPC biogenesis and NB progression.

## Discussion

**Figure 8:**
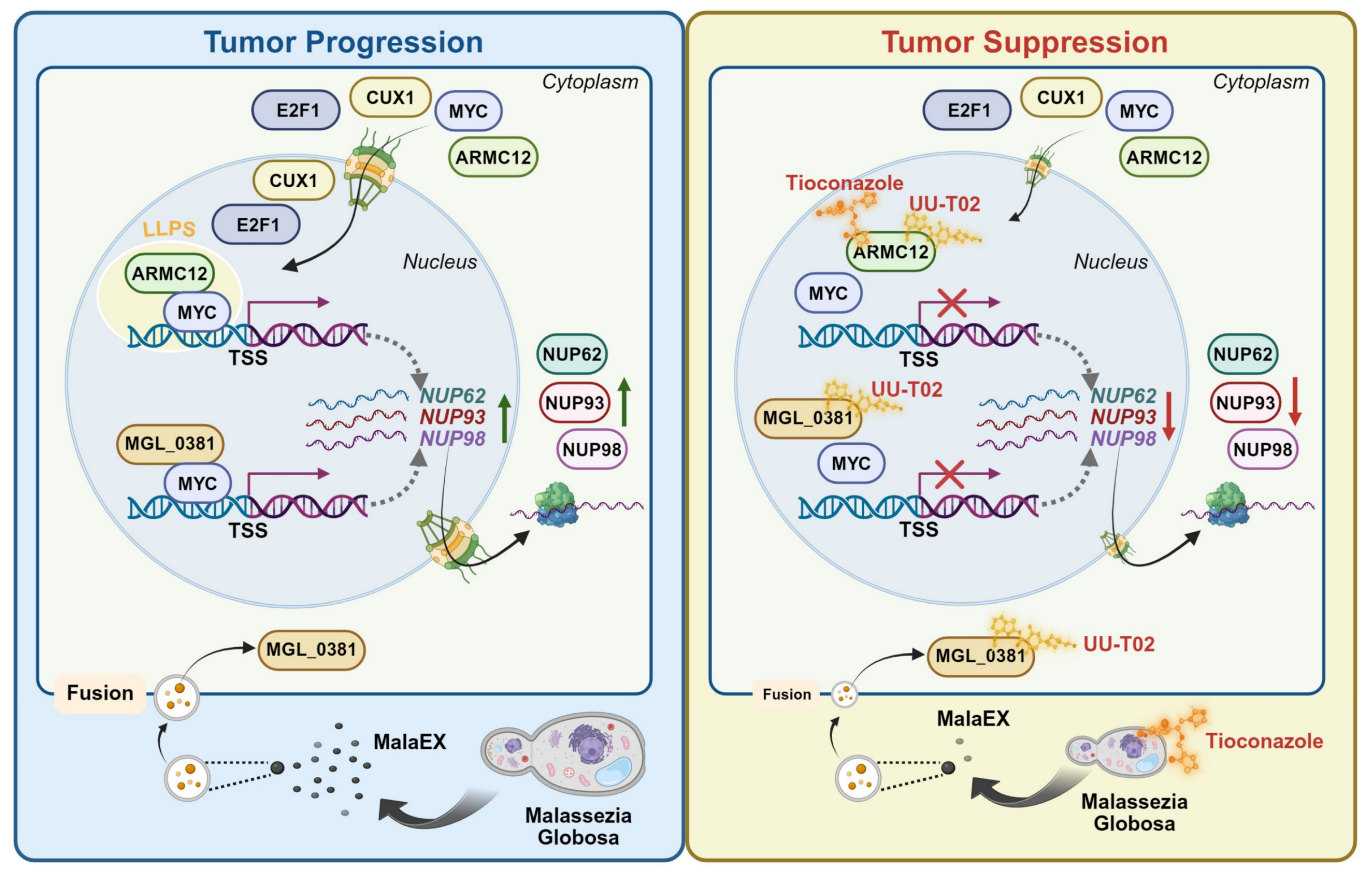
** Mechanisms underlying synergetic effects of AMRC12- and MGL_0381 in driving NPC biogenesis and progression of NB.** As a co-factor, AMRC12 binds with and activates transcription factor MYC in liquid condensates to facilitate target gene (*NUP62*, *NUP93*, and *NUP98*) expression, resulting in increase of NPC biogenesis and NB progression. In addition, as the protein content of extracellular vesicles (MalaEX) of *M. globosa*, MGL_0381 also physically interacts with and activates MYC protein to drive these target gene expressions. Meanwhile, anti-fungi tioconazole (TCZ) was able to block the interaction between ARMC12 and MYC, while UU-T02 binds with AMRC12 or MGL_0381 to suppress their interaction with MYC. Administration of both TCZ and UU-T02 exerts synergetic effects in repressing MYC transactivation, resulting in inhibition of NPC biogenesis and progression of NB.

The MYC family of transcription factors (MYC, MYCN, L-MYC) serves as pivotal oncogenic drivers in neuroendocrine tumors, such as cancers of the lung, pancreas, gastrointestinal tract, prostate, and NB 36. MYC protein binds E-box sequences (CACGTG) in target gene promoters, priming chromatin for active transcription 37. The *MYC* amplifications is noted in 15-20% of individuals diagnosed with small-cell lung cancer, and implicated in chemoresistance, tumor progression, or poor survival 38. In *MYC*-hyperactive tumors, MYC cooperates with E2F at promoters to amplify transcriptional output, accelerating cell cycle progression and proliferation 39. *MYC* promotes uncontrolled proliferation of tumor cells by activating cyclin-dependent kinase 4 in cell cycle S-phase entry 40 and fuels metabolic reprogramming via aerobic glycolysis or biosynthetic intermediate production 41. *MYC* also facilitates ribosomal DNA transcription by enhancing RNA polymerase I preinitiation complex assembly 42, 43. Proteomic studies reveal 336 high-confidence MYC partners, such as acetyltransferases, RNA processing factors, and ribosome biogenesis machinery, underscoring its multifaceted role in oncogenesis 44. Actually, the activity of MYC is regulated by binding partners, such as bromodomain containing 4 (BRD4) 45, non-POU domain containing octamer binding 21, or lamin A/C 16. Meanwhile, targeting *MYC* expression or activity is a potential therapeutic strategy for tumors. For example, MYCi975 is able to destabilize MYC via Helix-Loop-Helix domain binding and T58 phosphorylation 46, while Omomyc, a dominant-negative mini-protein, displaces MYC from E-boxes to suppress gene transcription 47. BET inhibitors are able to attenuate MYC activity by blocking BRD4-mediated chromatin remodeling 48. Herein, we discover that *MYC* facilitates the expression of *NUP62*, *NUP93*, and *NUP98*, resulting in elevation of NPC biogenesis, growth, and invasion capability of NB cells. In addition, our data demonstrate that ARMC12 engages with MYC within liquid-liquid phase-separated condensates to potentiate its transactivating function. This ARMC12-MYC interplay propels tumor growth and enhances aggressiveness (Figure 8), underscoring the pivotal role of the *ARMC12*/*MYC* axis in driving NB progression.

Armadillo repeat-containing proteins (ARMCs), a conserved eukaryotic protein family characterized by tandem repeats of ARM domains, play pivotal roles in cell adhesion, signal transduction, cytoskeletal regulation, mitochondrial dynamics, and tumorigenesis 49. In current study, a functional relationship between ARMC12 and MYC activities is illuminated in NB. *ARMC12* is highly conserved in primates (99% identity in gorillas, 84% in mice), and regulates mitochondrial dynamics during spermiogenesis 50. Our previous evidences establish ARMC12 as a critical cofactor of RB binding protein 4 in enhancing polycomb repressive complex 2 activity, thus silencing tumor suppressive genes and promoting proliferation of NB cells 15. Canonical ARMCs such as β-catenin, plakoglobin, and plakophilin utilize their ARM domains for protein-protein interactions 51. We demonstrated that ARM2 domain of ARMC12 protein mediated its binding to MYC, leading to elevation of MYC transactivation in NB cells. In addition, ARMC12 promoted NPC biogenesis during NB progression, at least in part, through elevation of MYC activation.

Recent evidences show the colonization of fungi within cancer tissues 52. For instance, pancreatic neoplasms exhibit the significant elevation in fungal biomass relative to normal counterparts, with mycobiota composition dominated by *M. globosa*, *Aspergillus*, and *Saccharomyces cerevisiae* 24. Notably, *M. globosa*, a lipophilic basidiomycetous yeast traditionally linked to dermatological conditions, has emerged as a critical oncogenic commensal in multiple cancers 24, 53, 54. *M. globosa* is enriched in pancreatic ductal adenocarcinoma (PDAC), breast carcinomas, and gastric malignancies, and serves as a predictive biomarker for adverse clinical outcomes 24, 53, 54. In PDAC, *M. globosa* accelerates oncogenesis via mannose-binding lectin signaling, and its removal via antifungal therapy (e.g., amphotericin B) reduces tumor burden and improves prognosis of mice 24. Similarly, *M. globosa* colonization promotes breast cancer progression by inducing interleukin 17A-driven M2 macrophage polarization 53. Of note, *M. globosa* encodes enzymes (esterases, lipases, proteases) that disrupt epithelial barriers, degrade extracellular matrix, or amplify inflammatory cascades, which collectively drive cancer progression 55. In this study, we found that *M. globosa* colonized within NB tissues, and served as an oncogenic mediator to drive NB progression. As lipid-bound nanosized particles (10-400 nm) releasing from all domains of life (eukaryotes, bacteria, fungi, archaea), EVs serve as critical mediators of intercellular and cross-kingdom communication by transporting proteins, nucleic acids, lipids, or metabolites 56. Bacterial EVs deliver immunomodulatory signals to up-regulate programmed death-ligand 1 in pulmonary carcinoma via signaling cascade from Toll-like receptor 4 to nuclear factor kappa B 34. In fungi, EVs were first identified in *Cryptococcus neoformans* in 2007, and have since been characterized in 11 additional species, such as *Candida albicans*, *Aspergillus fumigatus*, and *Paracoccidioides brasiliensis* 56. Fungal EVs facilitate unconventional export of proteins or specie-specific cargo involved in stress response or signaling transduction 56, and regulate intraspecies interactions 56. Notably, *Malassezia* species produce EVs that influence microbial community dynamics and host-pathogen crosstalk 57. In this investigation, we employed mass spectrometry to identify MGL_0381 as a protein content within EVs of *M. globosa*. Of importance, MGL_0381 promoted NPC biogenesis and NB progression by facilitating *MYC* transactivation. Disruption of MGL_0381-MYC interaction suppressed the malignant behaviors of NB cells, which underscores the critical role of MGL_0381 in NB pathogenesis.

Tioconazole (TCZ), an antifungal drug, is able to occupy the active sites of autophagy-related proteases autophagy related 4A (ATG4A) or autophagy related 4B (ATG4B), and directly block their enzymatic activity 58, thus diminishing autophagic flux in cancer cells under stress conditions 58. These findings position TCZ as a promising candidate for tumor treatment. In this study, using a novel drug discovery platform that integrates computational docking, biochemical assays, and cellular reporter systems, we identified that TCZ bound with ARMC12 to disrupt its interaction with MYC in NB cells, while underlying alteration in spatial conformation of ARMC12 protein warrants further investigation. Especially, our evidences demonstrate that administration of TCZ and UU-T02 exerts synergetic effects to inhibit NPC biogenesis and progression of NB. Meanwhile, further studies are needed to explore their effects and alternative acting mechanisms for tumor treatment in immunocompetent mice.

## Conclusions

In conclusion, our study provides inaugural evidence that ARMC12/MYC-modulated transcriptional targets in NPC correlate with adverse clinical outcomes and function as critical drivers of NB pathogenesis. The mechanistic basis involves ARMC12, which functions within liquid-liquid phase-separated condensates to co-activate MYC, driving the subsequent up-regulation of *NUP62*, *NUP93*, and *NUP98*, elevation of NPC number, and tumor progression. As a protein content within EVs of *M. globosa*, MGL_0381 also facilitates MYC transactivation via physical interaction to drive NPC biogenesis. Targeting both ARMC12-MYC and MGL_0381-MYC interaction exerts synergetic inhibitory effects on NPC biogenesis and NB progression. Collectively, our study elucidates the transcriptional regulatory networks governing NPC biogenesis and NB progression. Specifically, we uncover the pivotal roles of a fungal-derived protein and *ARMC12*/*MYC* axis in these processes, thereby highlighting their potential as novel therapeutic targets in oncology.

## Supplementary Material

Supplementary figures and tables.

## Abbreviations

1,6-HD: 1,6-hexanediol
AGC: automatic gain control
ANOVA: analysis of variance
ARMC12: armadillo repeat containing 12
ARMCs: Armadillo repeat-containing proteins
ATCC: American Type Culture Collection
BiFC: bimolecular fluorescence complementation
ChIP: chromatin immunoprecipitation sequencing
ChIP-seq: chromatin immunoprecipitation sequencing
co-IP: co-immunoprecipitation
CRISP: clustered regularly interspaced short palindromic repeats
CUX1: cut like homeobox 1
DAPI: 4',6-diamidino-2-phenylindole
dCas9: deactivated Cas9
DDA: data-dependent acquisition
DSF: differential scanning fluorimetry
E2F1: E2F transcription factor 1
EVs: extracellular vesicles
FDA: Food and Drug Administration
FISH: fluorescence in situ hybridization
FPKM: fragments per kilobase of transcript per million mapped reads
FRAP: fluorescence recovery after photobleaching
GAPDH: glyceraldehyde-3-phosphate dehydrogenase
GEO: Gene Expression Omnibus
GNB: ganglioneuroblastoma
GSEA: gene set enrichment analysis
GST: glutathione S-transferase
HA: hemagglutinin
HH3: histone H3
IDR: intrinsically disordered region
INSS: International Neuroblastoma Staging System
IPZ: importazole
ITS: Internal Transcribed Spacer
LC-MS/MS: liquid chromatography-tandem mass spectrometry
LLPS: liquid-liquid phase separation
NB: neuroblastoma
NE: nuclear envelope
NPC: nuclear pore complex
NUP: nucleoporin
PAGE: polyacrylamide gel electrophoresis
PBS: phosphate-buffered saline
PDAC: pancreatic ductal adenocarcinoma
PVDF: polyvinylidene fluoride
qPCR: quantitative PCR
qRT-PCR: quantitative RT-PCR
RNA-seq: RNA sequencing
SDS: sodium dodecyl sulfate
SEM: standard error of the mean
sgRNA: small guide RNA
shRNA: short hairpin RNA
sh-Scb: scramble shRNA
TCZ: tioconazole
TEM: transmission electron microscopy
