# Supplementary material for: Dual targeting of AMRC12 and Malassezia globosa disrupts MYC liquid condensates-driven nuclear pore complex biogenesis in neuroblastoma
Source: Theranostics. 2026 Jan 1;16(6):2866–86. doi: 10.7150/thno.120935 (PMC12775802; doi:10.7150/thno.120935)
Supplement: Supplementary file 1 — Supplementary figures and tables. [file thnov16p2866s1.pdf]

**Supporting Information**

# Dual targeting of AMRC12 and *Malassezia globosa* disrupts MYC liquid condensates-driven nuclear pore complex biogenesis in neuroblastoma

*Anpei Hu*, *Chunhui Yang*, *Zhijie Wang*, *Xiaolin Wang*, *Xinyue Li*, *Jiaying Qu*, *Shunchen Zhou*, *Bosen Zhao*, *Xiaojing Wang* *, *Liduan Zheng* *, *Qiangsong Tong* *

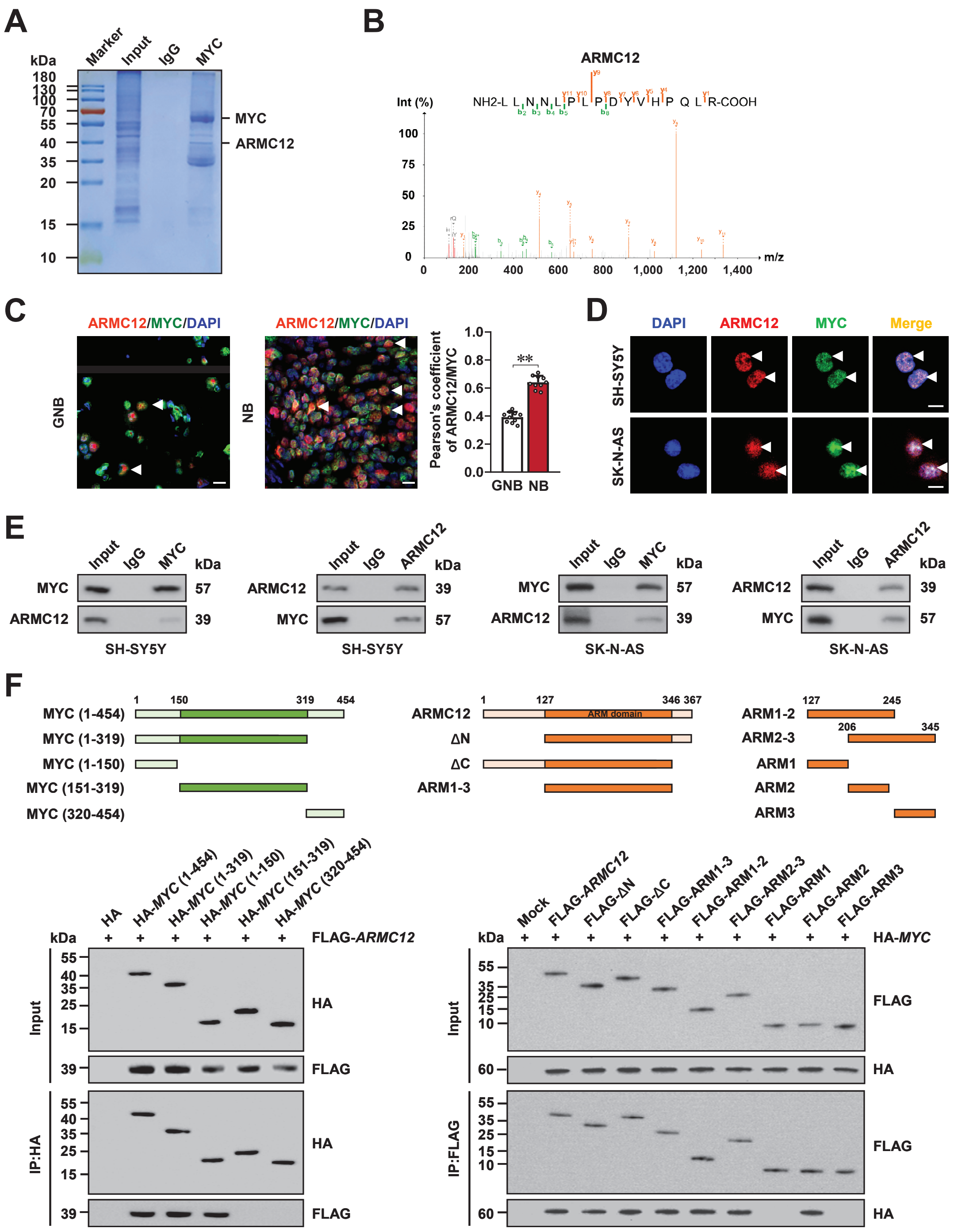

**Figure S1. ARMC12 interacts with MYC in NB.** (**A**) Coomassie blue staining showing the proteins pulled down by MYC-specific antibody from SH-SY5Y cell lystaes. (**B**) Mass spectrometry assay of ARMC12 peptide pulled down by MYC-specific antibody from SH-SY5Y cell lystaes. (**C**) Representative images (left panel) and quantification (right panel) of immunofluorescence assay revealing the co-localization of ARMC12 and MYC (arrowheads) in ganglioneuroblastoma (GNB) or NB specimens ($n$ = 5), with nuclei staining with DAPI. Scale bars: 50 μm. (**D**) Representative images of immunofluorescence assay showing co-localization of ARMC12 and MYC (arrowheads) in SH-SY5Y and SK-N-AS cells, with nuclei staining with DAPI. Scale bars: 10 μm. (**E**) Co-IP and western blot assays indicating endogenous interaction between ARMC12 and MYC in SH-SY5Y and SK-N-AS cells. (**F**) Co-IP and western blot assays indicating the protein levels of ARMC12 and MYC in SK-N-BE(2) cells transfected with FLAG-tagged *ARMC12* and HA-tagged *MYC* truncation constructs. Student's $t$ test compared the difference in **C**. ** $P < 0.01$. Data are shown as mean ± s.e.m. (error bars) or representative of three independent experiments in **A** and **C**-**F**.

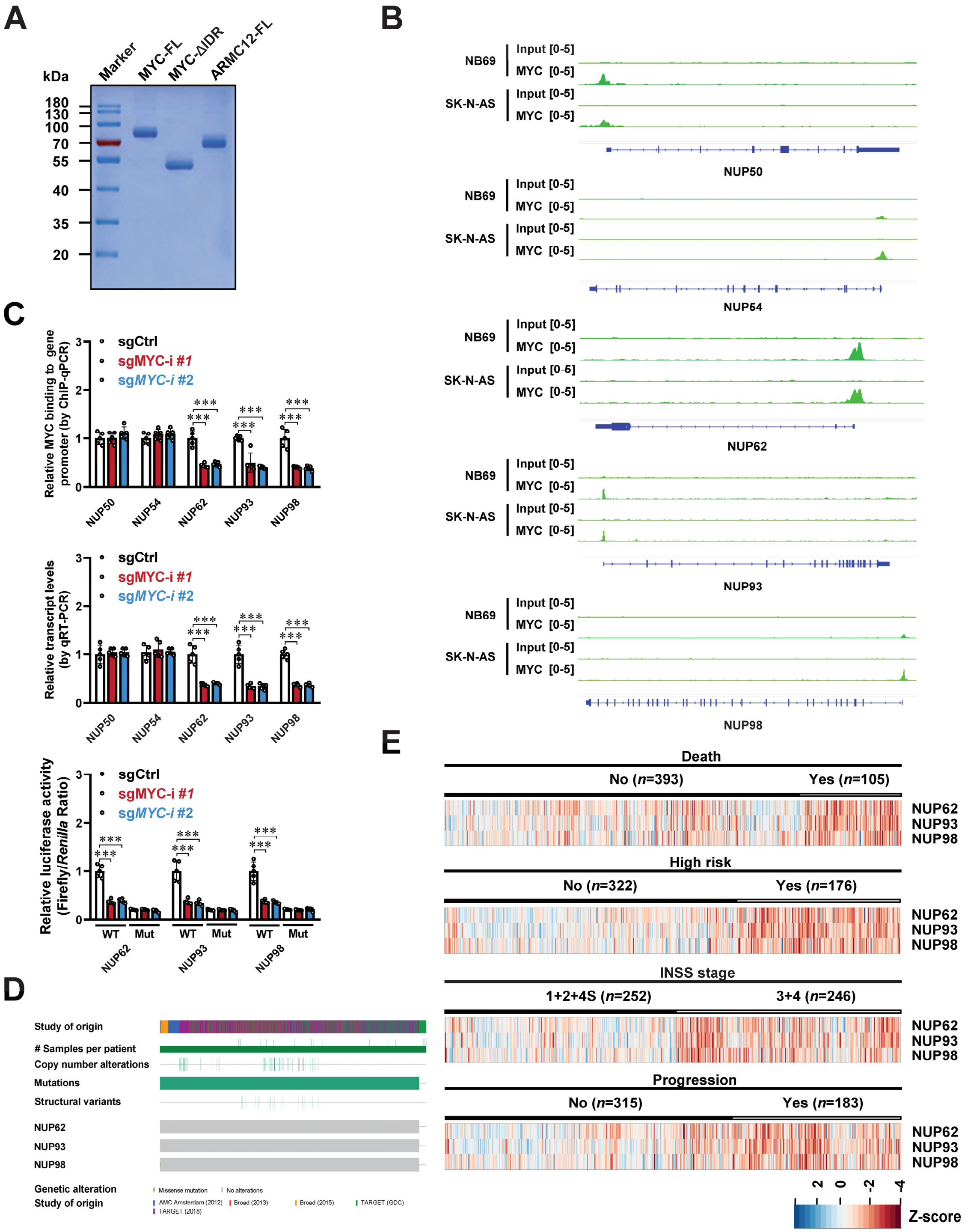


**Figure S2. MYC facilitates expression of NPC components in NB.** (**A**) Coomassie blue staining showing the purity of recombinant full-length (FL) or IDR-deficient (ΔIDR) MYC-EGFP and ARMC12-mCherry. (**B**) Mining of a public ChIP-seq dataset (GSE138295) revealing endogenous enrichment of MYC on promoter regions of *NUP50*, *NUP54*, *NUP62*, *NUP93*, and *NUP98* in NB cells. (**C**) ChIP-qPCR (normalized to input, $n$ = 5), real-time qRT-PCR (normalized to *β-actin*, $n$ = 5), and dual-luciferase ($n$ = 5) assays indicating the MYC enrichment, transcript levels, and activity of promoter luciferase reporter containing wild-type (WT) or mutant (Mut) MYC binding sites of *NUP62*, *NUP93*, or *NUP98* in SH-SY5Y stably transfected with control sgRNA (sgCtrl), sg*MYC*-i #1, or sg*MYC*-i #2. (**D**) Mining of cBioportal database (https://www.cbioportal.org/) showing no missense mutation, nonsense mutation, insertion, or deletion of *NUP62*, *NUP93*, and *NUP98* gene in 2001 NB cases. (**E**) Heatmap indicating the expression of *NUP62*, *NUP93*, and *NUP98* gene in 498 NB cases (GSE62564) with different status of death, high risk, INSS stage, or progression. One-way ANOVA compared the difference in **C**. *** $P < 0.001$. Data are shown as mean ± s.e.m. (error bars) or representative of three independent experiments in **C**.

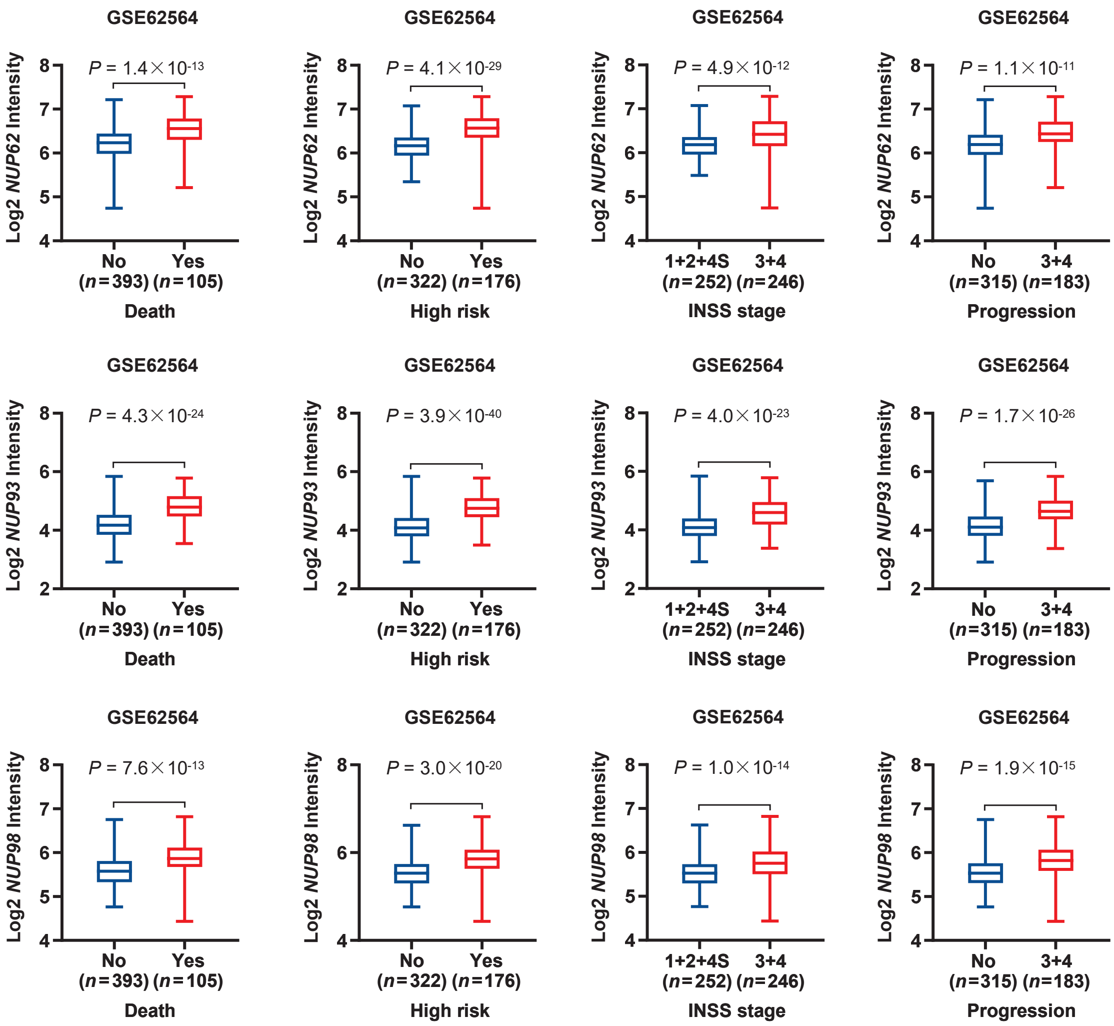


**Figure S3. Expression profiles of NPC components in NB.** Box plot indicating the expression levels of *NUP62*, *NUP93*, and *NUP98* in 498 NB cases (GSE62564) with different status of death, high risk, INSS stage, or progression. Mann-Whitney U test (for non-normal distributions) compared the difference.

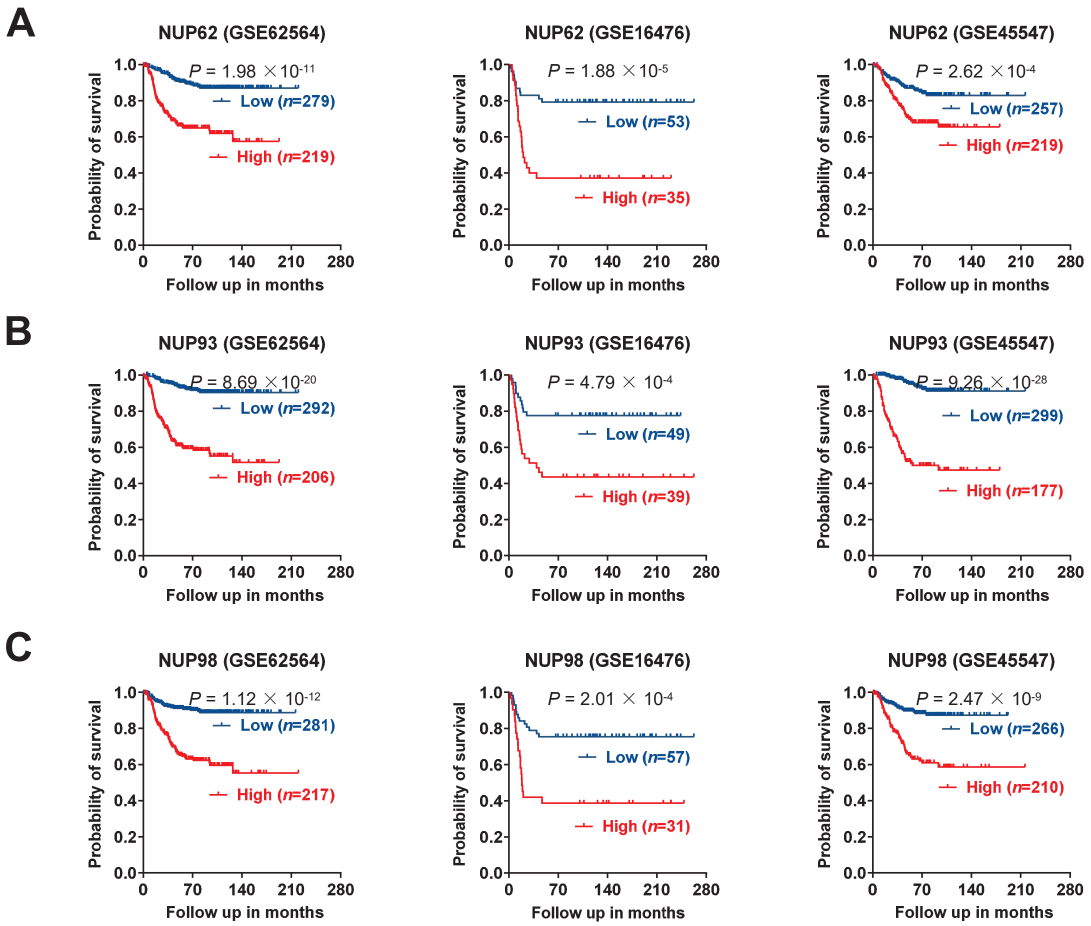


**Figure S4. Prognostic values of NPC components in NB.** Kaplan-Meier curve showing overall survival of 498 (GSE62564), 88 (GSE16476), and 649 (GSE45547) NB patients with high or low expression of *NUP62* (**A**, cutoff values = 6.34, 8.19 and 14.38), *NUP93* (**B**, cutoff values = 4.44, 6.61 and 10.89), or *NUP98* (**C**, cutoff values = 5.69, 6.78 and 10.98). Log-rank test for survival comparison.

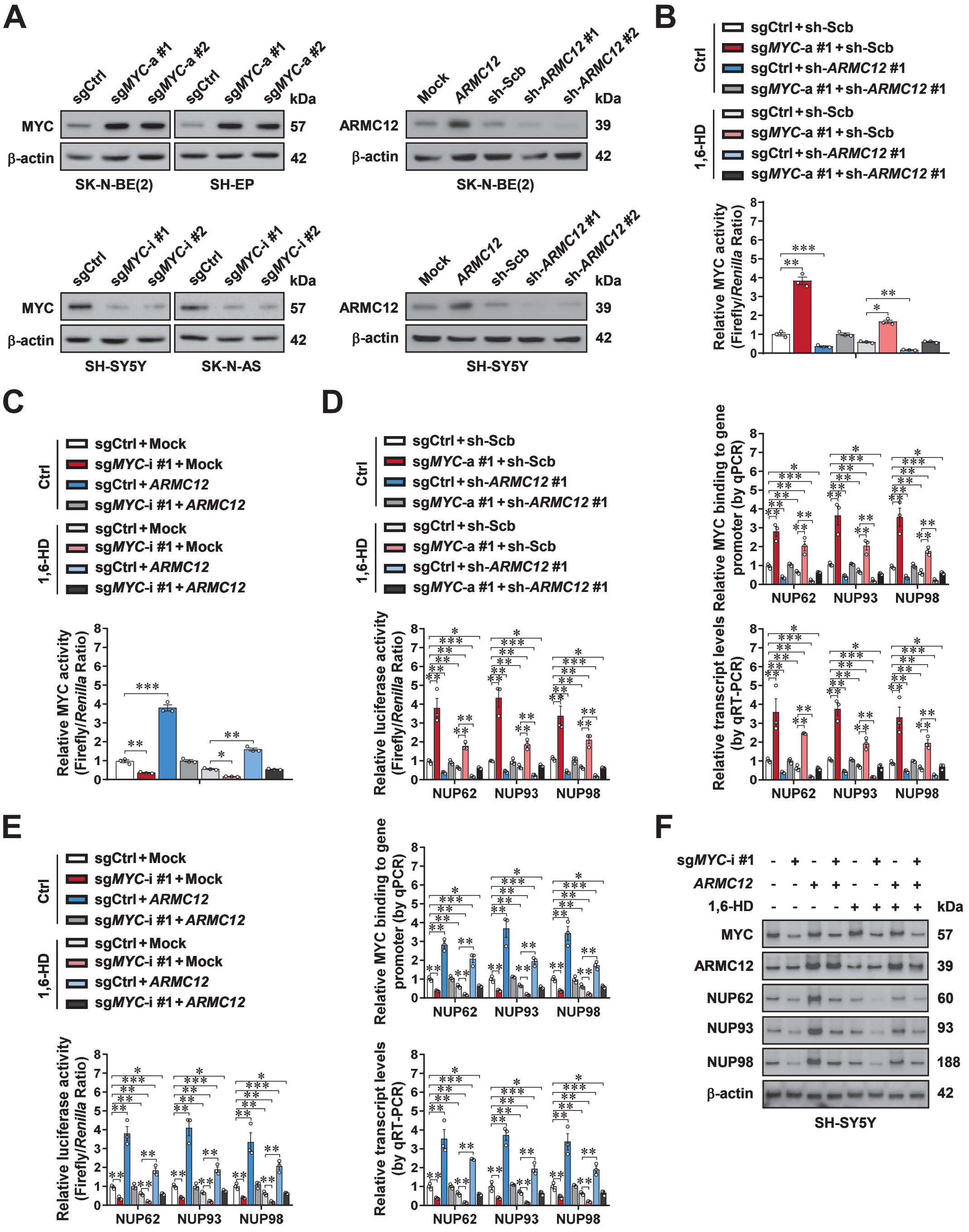

**Figure S5. ARMC12 facilitates MYC-medicated gene expression and NPC biogenesis in NB cells.** (**A**) Western blot assay showing the levels of MYC or AMRC12 in NB cells stably transfected with control sgRNA (sgCtrl), sg*MYC*-a #1, sg*MYC*-a #2, sg*MYC*-i #1, sg*MYC*-i #2, empty vector (mock), *ARMC12*, scramble shRNA (sh-Scb), sh-*ARMC12* #1, or sh-*ARMC12* #2. (**B** and **C**) Dual-luciferase assay indicating the MYC activity in SH-N-BE(2) cells stably transfected with sgCtrl, sg*MYC*-a #1, sg*MYC*-i #1, sh-Scb, sh-*ARMC12* #1, mock, or *ARMC12*, and those treated with vehicle or 10% 1,6-HD ($n$ = 3). (**D-F**) ChIP-qPCR (D, E, normalized to input, $n$ = 3), dual-luciferase (D, E, $n$ = 3), and real-time qRT-PCR (D, E, normalized to *β-actin*, $n$ = 3), and western blot (F) assays indicating the MYC enrichment, promoter activity, transcript levels, and protein expression of *NUP62*, *NUP93*, or *NUP98* in SH-N-BE(2) cells stably transfected with sgCtrl, sg*MYC*-a #1, sg*MYC*-i #1, sh-Scb, sh-*ARMC12* #1, mock, or *ARMC12*, and those treated with vehicle or 10% 1,6-HD. One-way ANOVA compared the difference in **B**-**E**. * $P$ < 0.05, ** $P$ < 0.01, *** $P$ < 0.001. Data are shown as mean ± s.e.m. (error bars) or representative of three independent experiments in **A**-**F**.

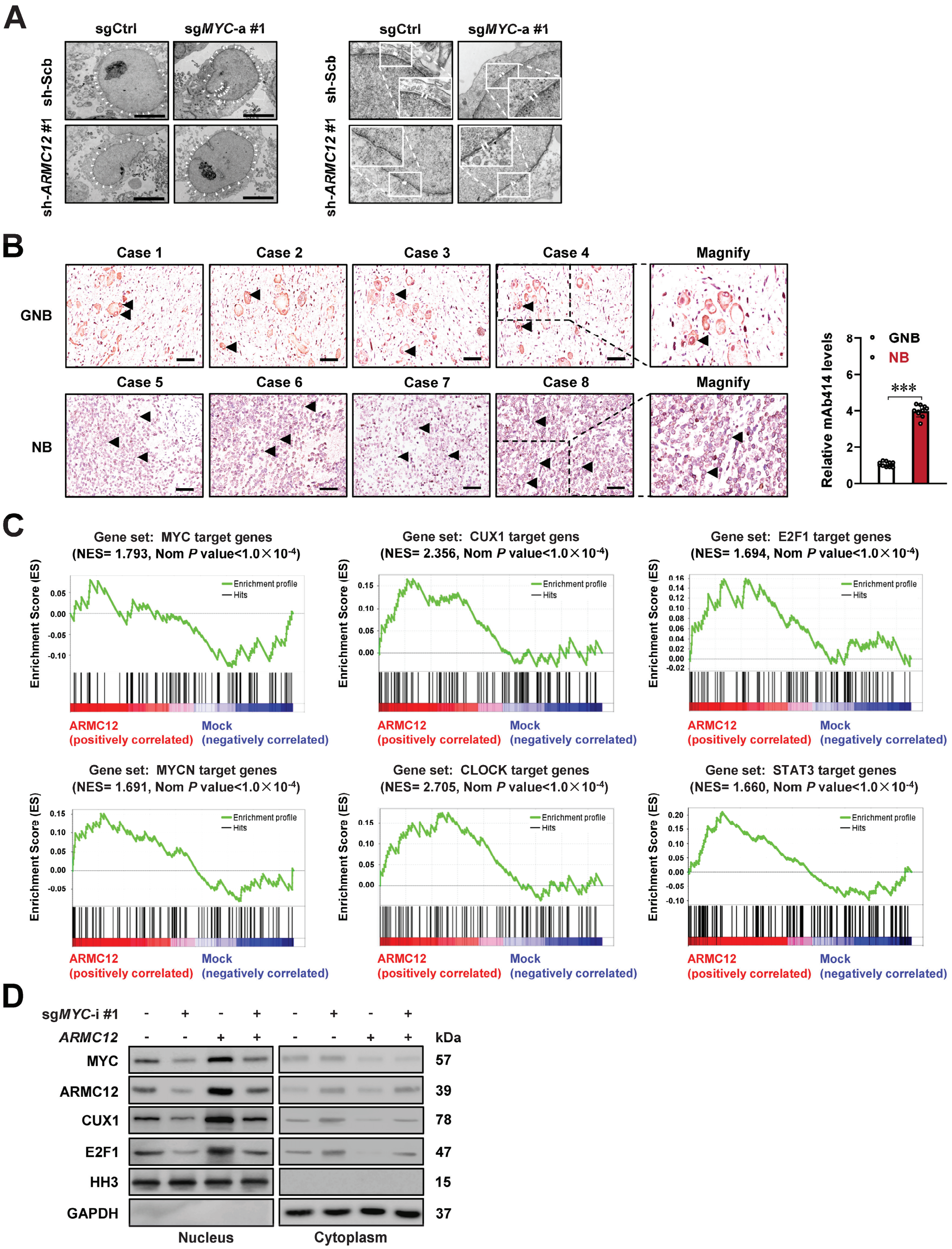


**Figure S6. ARMC12 cooperates with MYC to facilitate nuclear transport of transcriptional regulators in NB cells.** (**A**) Transmission electron microscopy indicating the NPC numbers and nuclear envelope (NE) spacing (arrowheads) in SH-N-BE(2) cells stably transfected with control sgRNA (sgCtrl), sg*MYC*-a #1, scramble shRNA (sh-Scb), or sh-*ARMC12* #1, and those treated with vehicle or 10% 1,6-HD. (**B**) Representative images (left panel) and quantification (right panel) of immunohistochemistry using mAb414 indicating the expression of NPC at nuclear envelope (arrowheads) in GNB and NB tissues ($n$ = 10). Scale bars: 100 μm. (**C**) GSEA of transcriptional regulators for altered genes in SK-N-BE(2) cells stably transfected with empty vector (mock) or *ARMC12*. (**D**) Western blot assay showing the nuclear or cytoplasmic distribution of MYC, ARMC12, CUX1, or E2F1 in SH-SY5Y cells stably transfected with control sgRNA (sgCtrl), sg*MYC*-i #1, mock, or *ARMC12*. Student's $t$-test compared the difference in **B**. *** $P < 0.001$. Data are shown as mean ± s.e.m. (error bars) or representative of three independent experiments in **A**, **B** and **D**.

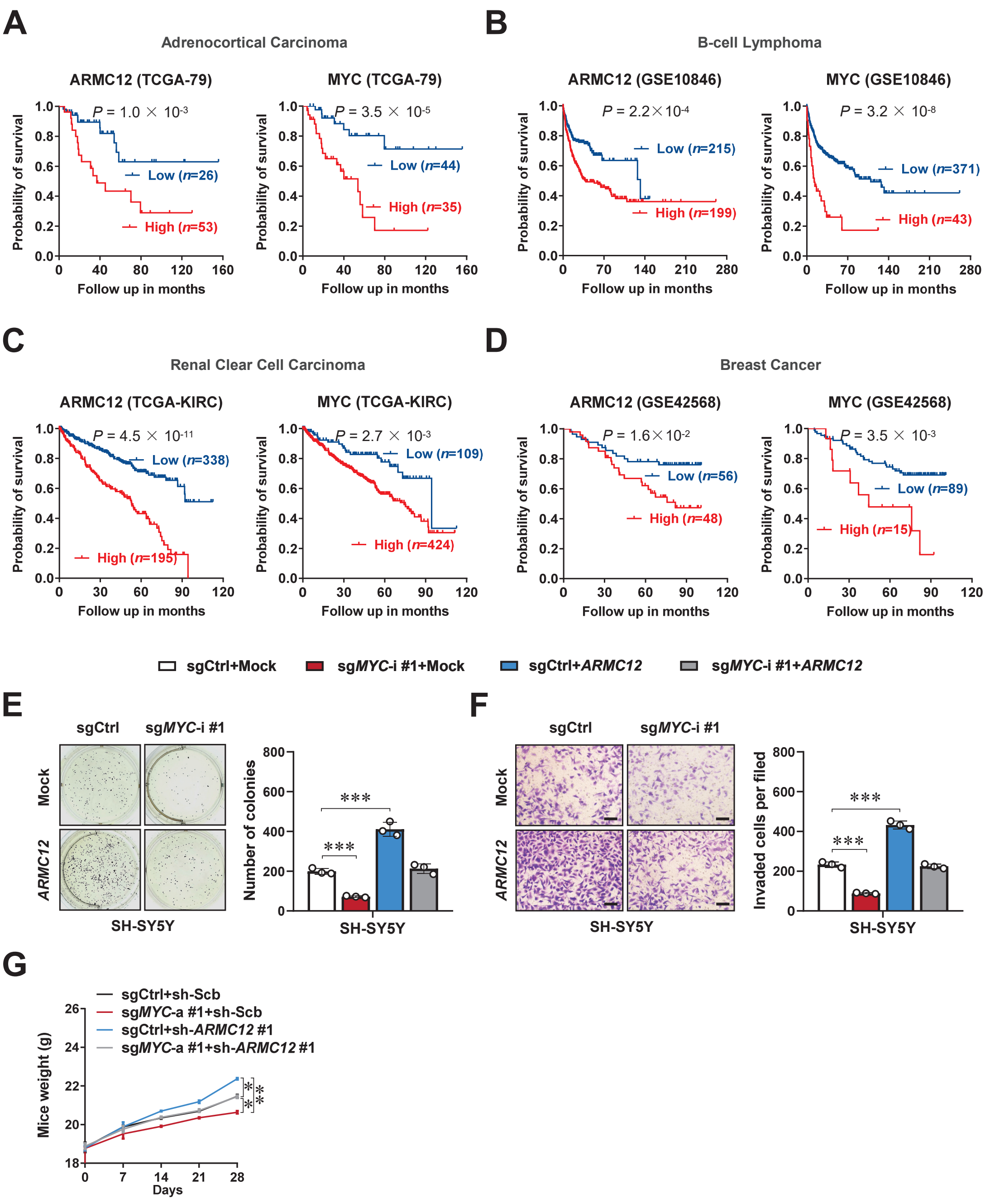


**Figure S7. ARMC12 cooperates with MYC to exert oncogenic functions.** (**A-D**) Kaplan-Meier curve showing overall survival of patients suffering from adrenocortical carcinoma (A, cutoff values = 4.08 and 8.84), B-cell lymphoma (B, cutoff values = 4.45 and 10.31), renal clear cell carcinoma (C, cutoff values = 2.44 and 10.22), or breast cancer (D, cutoff values = 3.83 and 8.96) with high or low expression levels of *ARMC12* or *MYC*. (**E** and **F**) Representative images (left panel) and quantification (right panel) of soft agar (E) and Matrigel invasion (F) assays showing the growth and invasion of SH-SY5Y cells stably transfected with control sgRNA (sgCtrl), sg*MYC*-i #1, empty vector (mock), or *ARMC12* ($n$ = 3). Scale bars: 50 μm. (**G**) Body weight of nude mice receiving vein tail injection of SK-N-BE(2) cells stably transfected with sgCtrl, sg*MYC*-a #1, sh-Scb, or sh-*ARMC12* #1 ($n$ = 5 per group). Log-rank test for survival comparison in **A-D**. One-way ANOVA compared the difference in **E-G**. * $P < 0.05$, ** $P < 0.01$, *** $P < 0.001$. Data are shown as mean ± s.e.m. (error bars) or representative of three independent experiments in **E-G**.

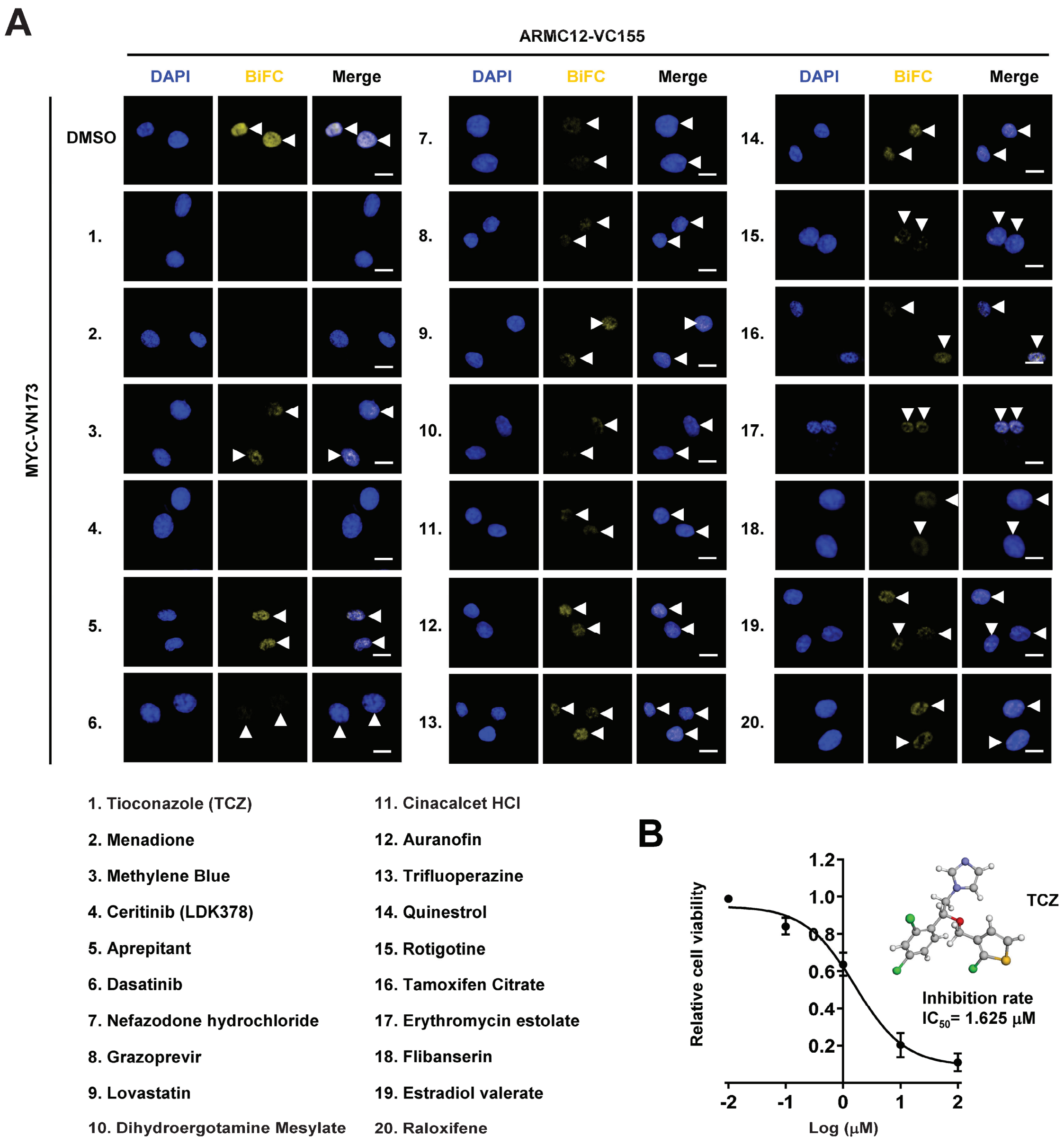


**Figure S8. Screening of inhibitors targeting ARMC12-MYC interaction.** (**A**) Representative images of BiFC assay showing the impact of indicated chemical agents on interaction between ARMC12 and MYC (arrowheads) in SH-SY5Y cells. (**B**) MTT colorimetric assay indicating the viability of SH-SY5Y cells treated with different doses of TCZ as indicated. Data are shown as mean ± s.e.m. (error bars) or representative of three independent experiments in **A** and **B**.

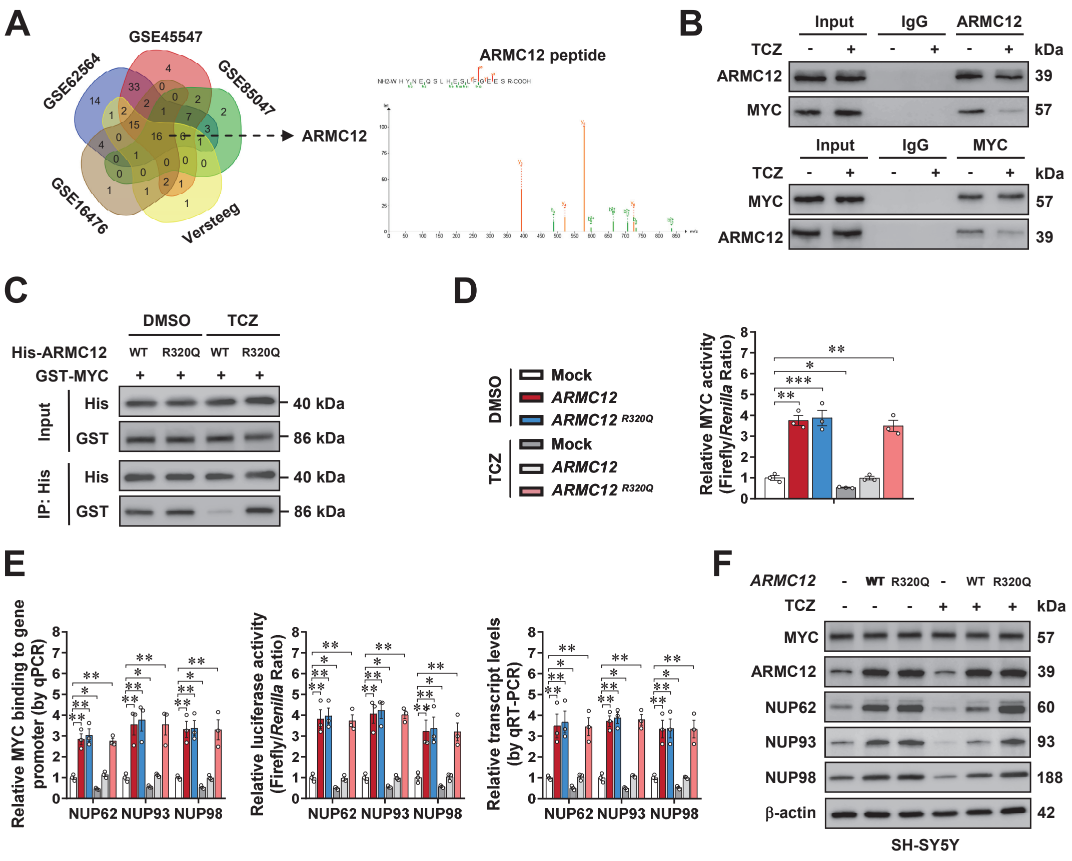

**Figure S9. TCZ binds with ARMC12 to disrupt its interaction with MYC.** (**A**) Venn diagram (left panel) and peptide in mass spectrometry (right panel) showing the identification of proteins within SH-SY5Y cell lysates binding to NHS magenetic beads covalently conjugated with TCZ (20 μM), and those consistently associated with survival of 498 (GSE62564), 649 (GSE45547), 283 (GSE85047), 122 (Versteeg), and 88 (GSE16476) NB patients. (**B**) Co-IP and western blot assays revealing the interaction between ARMC12 and MYC in SK-N-AS cells treated with DMSO or TCZ (20 μM). (**C**) Co-IP and western blot assay indicating the binding of wild-type or R320Q mutant His-tagged ARMC12 to GST-tagged MYC protein incubated with DMSO or TCZ (20 μM). (**D**) Dual-luciferase assay indicating the MYC activity in SH-SY5Y cells stably transfected with empty vector (mock), *ARMC12*, or *ARMC12* mutant (R320Q), and those treated with DMSO or TCZ (20 μM, $n$ = 3). (**E** and **F**) ChIP-qPCR (E, normalized to input, $n$ = 3), dual-luciferase (E, $n$ = 3), and real-time qRT-PCR (E, normalized to *β-actin*, $n$ = 3), and western blot (F) assays indicating the MYC enrichment, promoter activity, transcript levels, and protein expression of *NUP62*, *NUP93*, or *NUP98* in SH-SY5Y cells stably transfected with mock, *ARMC12*, or *ARMC12* mutant (R320Q), and those treated with DMSO or TCZ (20 μM). One-way ANOVA compared the difference in **D** and **E**. * $P < 0.05$, ** $P < 0.01$, *** $P < 0.001$. Data are shown as mean ± s.e.m. (error bars) or representative of three independent experiments in **B-F**.

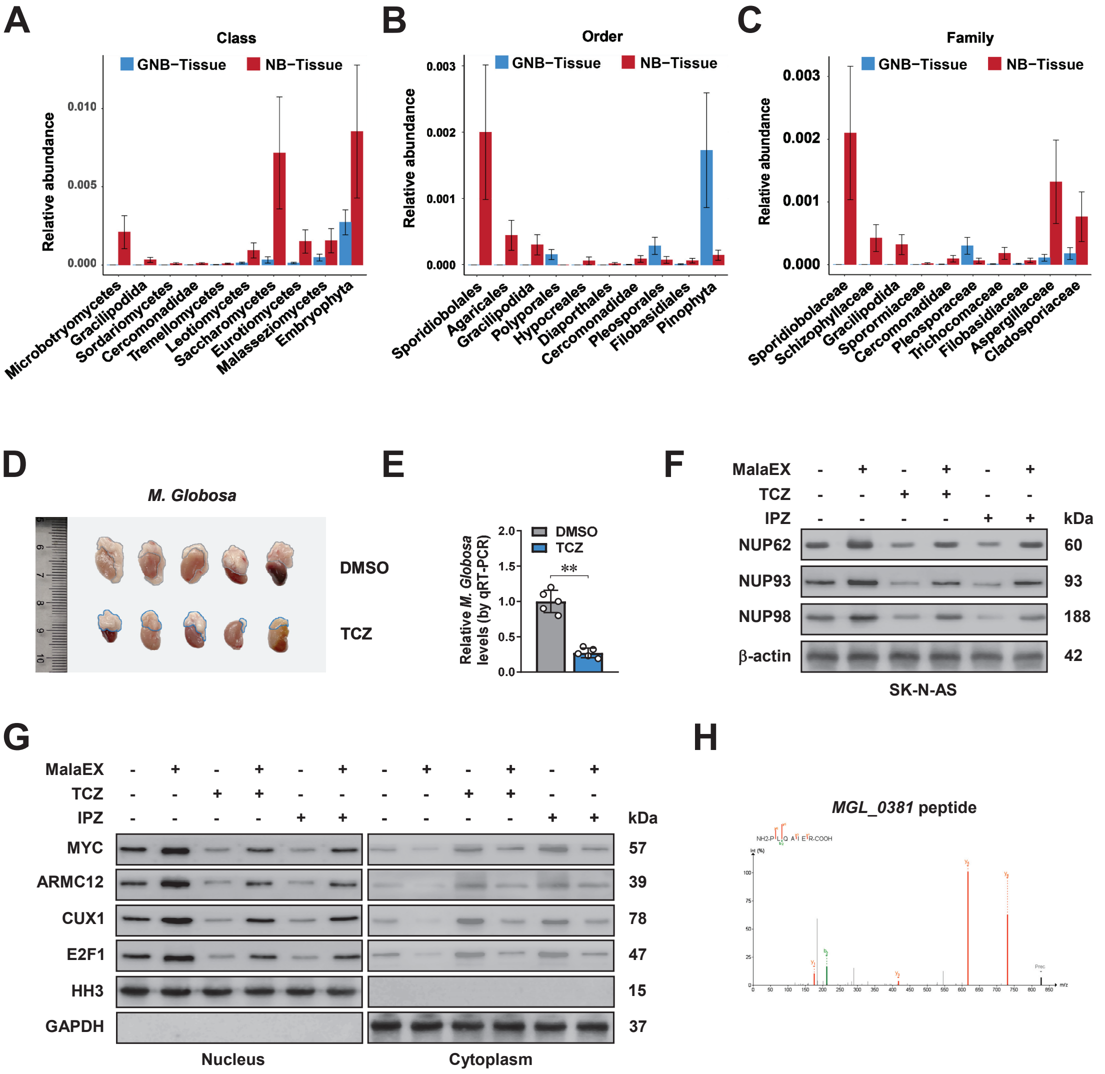


**Figure S10.** ***M. globosa* extracellular vesicles facilitates NPC biogeneis and tumorigenesis.** (**A-C**) Mycobiome analysis using 18S rRNA sequencing indicating the class (A), and order (B), or family (C) of fungi within GNB and NB tissues. (**D**) Representative images of xenograft tumors of SH-SY5Y cells under renal capsule of NSG mice receiving oral gavage of Amphotericin B, *M. globosa*, and intraperitoneal injection of TCZ (60 mg/kg/day). (**E**) Real-time qRT-PCR assay indicating the levels (normalized to *β-actin*, *n* = 5) of *M. globosa* 18S rRNA within xenograft tumors of SH-SY5Y cells under renal capsule of NSG mice receiving oral gavage of Amphotericin B, *M. globosa*, and intraperitoneal injection of TCZ (60 mg/kg/day). (**F**) Western blot assay showing the expression of NUP62, NUP93, and NUP98 in SK-N-AS cells treated with MalaEx, TCZ (20 μM), or UU-T02 (1.0 μM). (**G**) Western blot assay showing the nuclear or cytoplasmic distribution of MYC, ARMC12, CUX1, or E2F1 in SK-N-AS cells treated with MalaEx, TCZ (20 μM), or UU-T02 (1.0 μM). (**H**) Peptide of MGL_0381 in mass spectrometry assay of protein content within MalaEX. Student's *t*-test compared the difference in **E**. ** $P < 0.01$. Data are shown as mean ± s.e.m. (error bars) or representative of three independent experiments in **E-G**.

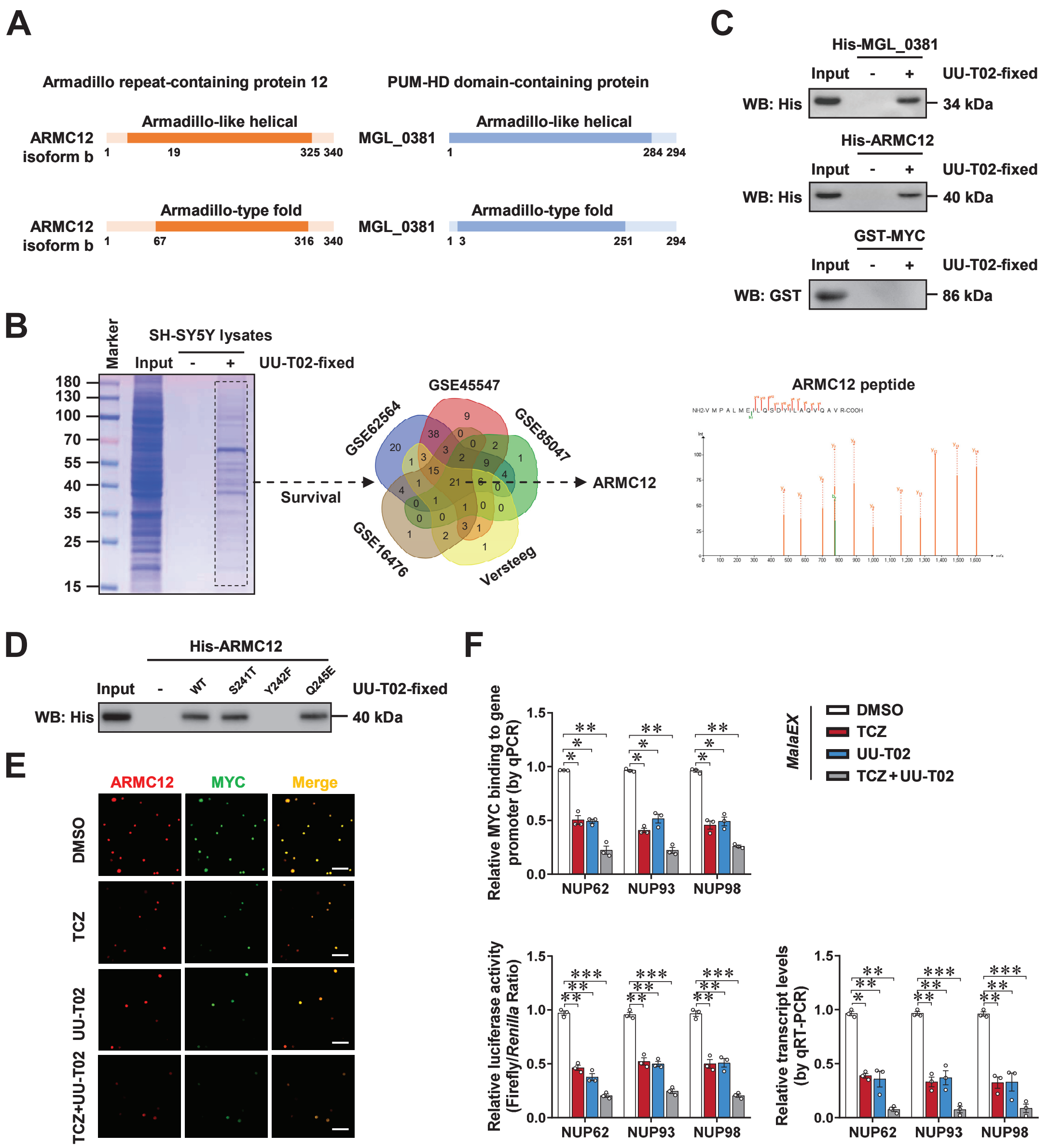


**Figure S11. TCZ and UU-T02 suppress liquid condensates and activity of MYC in NB cells.** **A**, Schematic illustration showing the domains of ARMC12 and MGL_0381 proteins. **B**, Coomassie blue staining (left panel) and Venn diagram (middle panel), and peptide in mass spectrometry (right panel) revealing the binding of proteins within SH-SY5Y cell lysates to NHS magenetic beads covalently conjugated with UU-T02 (1.0 μM), and those consistently associated with survival of 498 (GSE62564), 649 (GSE45547), 283 (GSE85047), 122 (Versteeg), and 88 (GSE16476) NB patients. **C**, Western blot assay indicating the affinity of recombinant His-tagged MGL_0381, His-tagged ARMC12, or GST-tagged MYC to NHS beads covalently conjugated with UU-T02 (1.0 μM). **D**, Western blot assay indicating the affinity of recombinant wild-type or mutant (S241T, Y242F, Q245E) ARMC12 protein to NHS beads covalently conjugated with UU-T02 (1.0 μM). **E**, Representative images indicating the liquid droplet formation of ARMC12-mCherry and MYC-EGFP *in vitro*, and those incubated with DMSO, TCZ (20 μM), or UU-T02 (1.0 μM). Scale bars: 10 μm. **F**, ChIP-qPCR (normalized to input, $n$ = 3), dual-luciferase ($n$ = 3), and real-time qRT-PCR (normalized to *β-actin*, $n$ = 3) assays indicating the MYC enrichment, promoter activity, and transcript levels of *NUP62*, *NUP93*, or *NUP98* in SK-N-AS cells treated with MalaEx, TCZ (20 μM), or UU-T02 (1.0 μM). One-way ANOVA compared the difference in **F**. * $P < 0.05$, ** $P < 0.01$, *** $P < 0.001$. Data are shown as mean ± s.e.m. (error bars) or representative of three independent experiments in **B-F**.

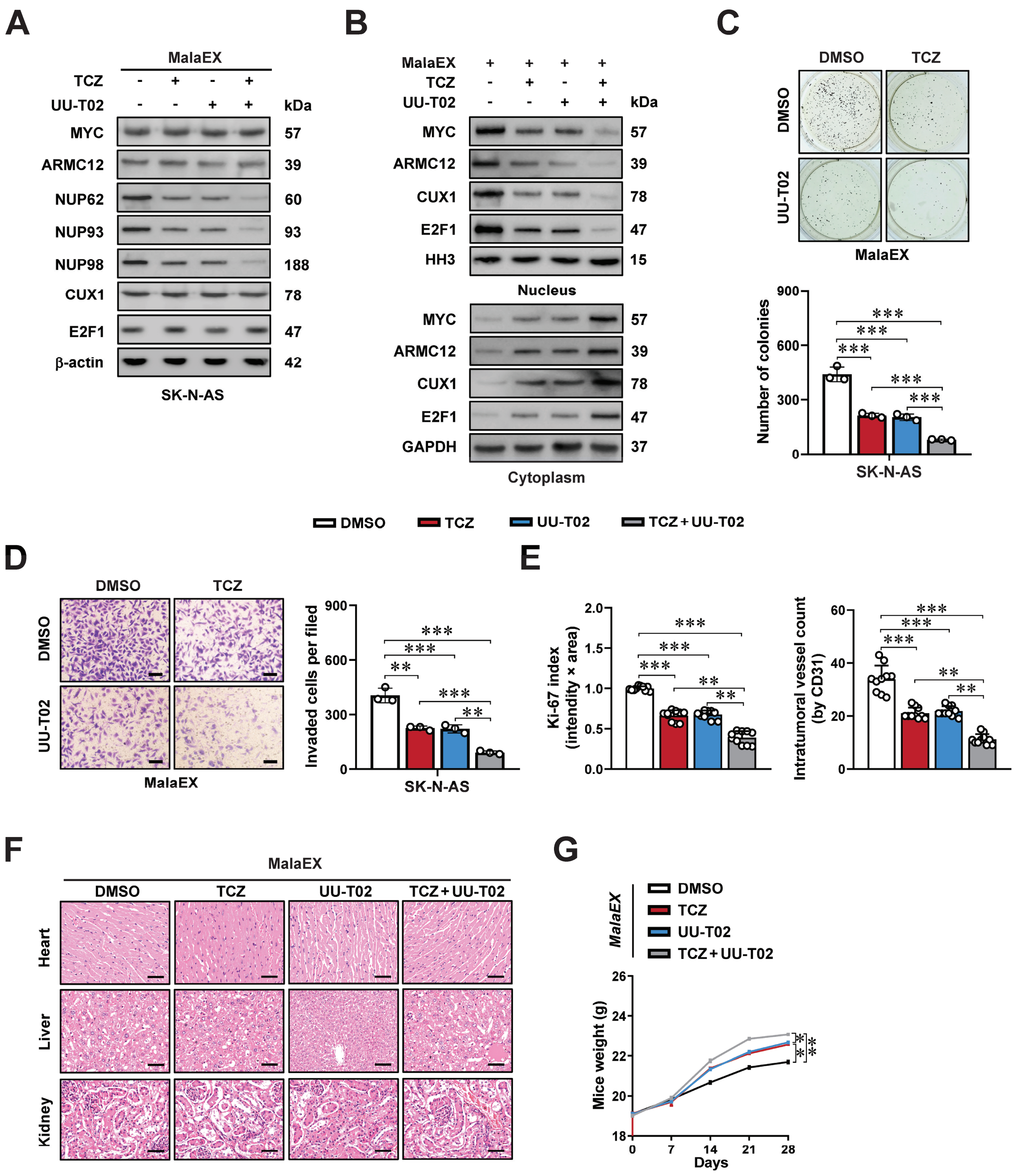


**Figure S12. Synergetic targeting MGL_0381 and AMRC12-facilitated MYC transactivation inhibits NPC biogenesis, growth, and invasion of NB cells. A** and **B**, Western blot assay indicating the protein expression of MYC, ARMC12, NUP62, NUP93, NUP98, CUX1, or E2F1 and nuclear or cytoplasmic distribution of MYC, ARMC12, CUX1, or E2F1 in SK-N-AS cells treated with MalaEx, TCZ (20 μM), or UU-T02 (1.0 μM). **C** and **D**, Representative images and quantification of soft agar (C) and Matrigel invasion (D) assays showing the growth and invasion of SK-N-AS cells treated with MalaEx, TCZ (20 μM), or UU-T02 (1.0 μM, $n$ = 3). Scale bars: 50 μm. **E**, Quantification of Ki-67 or CD31 immunostaining within subcutaneous xenograft tumors formed by SH-SY5Y cells in nude mice that received administration of MalaEx, TCZ (60 mg/kg/day), or UU-T02 (60 mg/kg/day, $n$ = 5 per group). **F**, HE staining showing the morphological changes of heart, liver, and kidney tissues of nude mice with SH-SY5Y-formed subcutaneous xenograft tumors and administration of MalaEx, TCZ (60 mg/kg/day), or UU-T02 (60 mg/kg/day). **G**, Body weight of nude mice treated with tail vein injection of SH-SY5Y cells in nude mice that received administration of MalaEx, TCZ (60 mg/kg/day), or UU-T02 (60 mg/kg/day, $n$ = 5 per group). One-way ANOVA compared the difference in **C-E** and **G**. * $P < 0.01$, ** $P < 0.01$, *** $P < 0.001$. Data are shown as mean ± s.e.m. (error bars) or representative of three independent experiments in **A-G**.

## Table S1 Primer sets used for RT-PCR, ChIP and probe

| Primer set | Primers | Sequence | Product size (bp) | Application |
|---|---|---|---|---|
| ALADIN | Forward | 5'-ACTGCCTTCATCCATCACCG-3' | 131 | RT-PCR |
| | Reverse | 5'-GCCGTCTTCACCCACTCAAA-3' | | |
| DDX19 | Forward | 5'-GCTTTTCTCCGCCACCTTTG-3' | 297 | RT-PCR |
| | Reverse | 5'-TCCACCATCATCTCCCCACT-3' | | |
| ELYS | Forward | 5'-CCTATTAGACGGCGTTACTG-3' | 170 | RT-PCR |
| | Reverse | 5'-CTATCAACCAAAACCCCTGA-3' | | |
| GLE1 | Forward | 5'-CCCTACGCAGTTCCGACAAA-3' | 129 | RT-PCR |
| | Reverse | 5'-CGTGCTCTACCACCCATCCA-3' | | |
| NDC1 | Forward | 5'-TGCTGCTGTCAGTGGTAATA-3' | 199 | RT-PCR |
| | Reverse | 5'-CTGTACTGGCCCTGGGTTAT-3' | | |
| RAE1 | Forward | 5'-GACCTCAGCAGTAACCAAGC-3' | 246 | RT-PCR |
| | Reverse | 5'-ATAGACAATCAGGCCCCTCT-3' | | |
| SEC13 | Forward | 5'-GAGGACATGATTCACGACG-3' | 114 | RT-PCR |
| | Reverse | 5'-GATAAGGATCTGCCCTCCA-3' | | |
| SEH1 | Forward | 5'-CAGTGGTCTTTGCAGCATGA-3' | 124 | RT-PCR |
| | Reverse | 5'-ATTCTCACATCTTTGGTCGC-3' | | |
| TPR | Forward | 5'-TCCACTGGCACTTTATCTAC-3' | 268 | RT-PCR |
| | Reverse | 5'-CACCTTGTCTCAATGTTGTC-3' | | |
| NUP37 | Forward | 5'-TAATGTCAGCACACTGGTG-3' | 100 | RT-PCR |
| | Reverse | 5'-TGAGGATAACTGGACCGAG-3' | | |
| NUP42 | Forward | 5'-CAATGGAGGAACAGGGTAAA-3' | 196 | RT-PCR |
| | Reverse | 5'-GACTGCTGCCAAATCCAAAT-3' | | |
| NUP43 | Forward | 5'-CCTGGCAGTCCTTCCTATAG-3' | 251 | RT-PCR |
| | Reverse | 5'-GGCTCATTTCCTTGTTGTCT-3' | | |
| NUP50 | Forward | 5'-ATAACCAGTGCCCCTCCCTTCG-3' | 207 | RT-PCR |
| | Reverse | 5'-GGCGGCCAACTGCTTGTGATAG-3' | | |
| NUP53 | Forward | 5'-CGATGACTCTTGGGTGACTG-3' | 245 | RT-PCR |
| | Reverse | 5'-CACTGCTTTCCATAACACTT-3' | | |
| NUP54 | Forward | 5'-TTGGCACAACAACGGGAACT-3' | 177 | RT-PCR |
| | Reverse | 5'-CAGAAAGAGCACTCGCAGTA-3' | | |
| NUP58 | Forward | 5'-GCAGTACAGGCAGCAGATTG-3' | 288 | RT-PCR |
| | Reverse | 5'-GGAGTGGGTCCAGTAGTAAC-3' | | |
| NUP62 | Forward | 5'-GGGGAACTGGATTTTCTTTGGG-3' | 131 | RT-PCR |
| | Reverse | 5'-GCTCGATATGGCATTAGTGAGG-3' | | |
| NUP75 | Forward | 5'-TGAAAGCCGTCCGCAACAAT-3' | 238 | RT-PCR |
| | Reverse | 5'-GCAAAACGCTTCTCCCCGTA-3' | | |
| NUP88 | Forward | 5'-AAAACCAGAGTCCAACCGAA-3' | 233 | RT-PCR |
| | Reverse | 5'-AAACAGGGGTGGATTTATGC-3' | | |
| NUP93 | Forward | 5'-TACTCAAGAAAGCGAGCCAAGC-3' | 157 | RT-PCR |
| | Reverse | 5'-AGCACAAAGGTCCACCAGGTTA-3' | | |
| NUP98 | Forward | 5'-CAGTTTGTGGGTAGCCAGTCAG-3' | 207 | RT-PCR |
| | Reverse | 5'-AAGATGGATAGCCAGGGAGCGT-3' | | |
| NUP107 | Forward | 5'-AGCGTTATTTCAGAGGGATT-3' | 163 | RT-PCR |
| | Reverse | 5'-CTGCCGTTGTTTTAAGGTAT-3' | | |
| POM121 | Forward | 5'-TTGGAGGACAAGAGCGATGC-3' | 283 | RT-PCR |
| | Reverse | 5'-GCGGCCCTGACTGTGATAAA-3' | | |
| NUP133 | Forward | 5'-AAGCTTTGGACTTGTTGGA-3' | 110 | RT-PCR |
| | Reverse | 5'-AGAACTGGACCAGTTATCT-3' | | |
| NUP153 | Forward | 5'-TGAAGCGAGCCCTTACATTG-3' | 299 | RT-PCR |
| | Reverse | 5'-TCCCTCGGGTTTCTTGAACT-3' | | |
| NUP155 | Forward | 5'-CACTGCTGCCTGTGATAGAG-3' | 210 | RT-PCR |
| | Reverse | 5'-ATACCATGAGACGGTGTTCC-3' | | |
| NUP160 | Forward | 5'-TTATGGCCTAGCAATCCTGG-3' | 103 | RT-PCR |
| | Reverse | 5'-GACACCAGGGATGTAGCAGT-3' | | |
| NUP188 | Forward | 5'-GGTCAGGGTATGTCAAGTCA-3' | 164 | RT-PCR |
| | Reverse | 5'-ACGCACCACAGAGTCAGTCA-3' | | |
| NUP205 | Forward | 5'-CTTCCAAGTGCAGATAGTGT-3' | 223 | RT-PCR |
| | Reverse | 5'-GTAGCTCAGCTTTTAGGACA-3' | | |
| NUP210 | Forward | 5'-TGGATGGACCCGAAAAGGTT-3' | 269 | RT-PCR |
| | Reverse | 5'-AGAGTTGTCGTGGAAGTGGA-3' | | |
| NUP214 | Forward | 5'-GTCATTCCTTGTCCTCCGTT-3' | 280 | RT-PCR |
| | Reverse | 5'-CAGATGCTGCCAGCACTAAA-3' | | |
| NUP358 | Forward | 5'-TTCTACACCGTCTCCTACCA-3' | 184 | RT-PCR |
| | Reverse | 5'-AGGGGATGCTGAGTTACTGC-3' | | |
| M. globosa | Forward | 5'-GCTTCTTAGAGGGACTATTGG-3' | 229 | RT-PCR |
| | Reverse | 5'-GCTGATGACTCACGCTTACT-3' | | |
| β-actin | Forward | 5'-TGCCCATCTACGAGGGGTATG-3' | 156 | RT-PCR |
| | Reverse | 5'-TCTCCTTAATGTCACGCACGATTT-3' | | |
| NUP50 | Forward | 5'-GGACTCGTTCCCCTGCTCTACA-3' | 109 | ChIP |
| | Reverse | 5'-ACCCCTCGTTCGCTGACATTTG-3' | | |
| NUP54 | Forward | 5'-AACATCGTGAAACCCTGTCTCT-3' | 286 | ChIP |

| | | | | |
|---|---|---|---|---|
| | Reverse | 5'-CTCTTGACCTCGTGATACGCCC-3' | | |
| NUP62 | Forward | 5'-TTCGGCCCACCGTACTACTTCT-3' | 133 | ChIP |
| | Reverse | 5'-CGCTTACCTCTTCTCCAACTGC-3' | | |
| NUP93 | Forward | 5'-GCCACGGTGTTGCCAGTTCCTA-3' | 146 | ChIP |
| | Reverse | 5'-ACGGACCTCGCCTTTCCCACTT-3' | | |
| NUP98 | Forward | 5’-ACTCTTTGGACAGACAGACCTT-3’ | 286 | ChIP |
| | Reverse | 5’-AACTATTCGACCCTTTCCTTGC-3’ | | |
| M. globosa | Forward | 5’-AATGAAGTATCGCCAGCACGAG-3’ | NA | Probe |

ALADIN, alacrima-achalasia-adrenal insufficiency neurologic disorder protein; DDX19, DEAD-box helicase 19; ELYS, Embryonic large molecule derived from Yolk sac; GLE1, GLE1 RNA export mediator homolog; NDC1, nuclear division cycle 1 homolog; RAE1, ribonucleic acid export 1; SEC13, SEC13 homolog, nuclear pore and COPII coat complex component; SEH1, SEH1 homolog; TPR, translocated promoter region, nuclear basket protein; NUP37, nucleoporin 37; NUP42, nucleoporin 42; NUP43, nucleoporin 43; NUP50, nucleoporin 50; NUP53, nucleoporin 53; NUP54, nucleoporin 54; NUP58, nucleoporin 58; NUP62, nucleoporin 62; NUP75, nucleoporin 75; NUP88, nucleoporin 88; NUP93, nucleoporin 93; NUP98, nucleoporin 98; NUP107, nucleoporin 107; POM121, POM121 transmembrane nucleoporin; NUP133, nucleoporin 133; NUP153, nucleoporin 153; NUP155, nucleoporin 155; NUP160, nucleoporin 160; NUP188, nucleoporin 188; NUP205, nucleoporin 205; NUP210, nucleoporin 210; NUP214, nucleoporin 214; NUP358, nucleoporin 358; M. globosa, Malassezia globosa; ChIP, chromatin immunoprecipitation.

## Table S2 Primer sets used for constructs

| Primer set | Primers | Sequence |
|---|---|---|
| pLenti-ARMC12 | Forward | 5'-CGCGGATCCATGGGCAAGAGCATCCCCCAATAC-3' |
| | Reverse | 5'-GCGCACCGGTTTCCGTGTTTTTAAAGTAGGAACG-3' |
| pET28a-ARMC12 | Forward | 5'-GTGGACAGCAAATGGGTCGCGGATCCATGGGCAAGAGCATCCCCCA-3' |
| | Reverse | 5'-GGTGGTGGTGGTGCTCGAGTGCGGCCGCTTCCGTGTTTTTAAAGTAGG-3' |
| pLenti-ARMC12 R320Q | Forward | 5'-CTGGCAGACCAACTACTTGCCCTGGTCATCCACCCTGAGGAA-3' |
| pET28a-ARMC12 R320Q | Reverse | 5'-GGGCAAGTAGTTGGTCTGCCAGTCGGGACTCTTCCCCAAAGA-3' |
| pET28a-ARMC12 S241T | Forward | 5'-CGACTGCTGACCTACCTGGCACAGAAGAATGACCTTCTCTAT-3' |
| | Reverse | 5'-GTGCCAGGTAGGTCAGCAGTCGTACGGCTTGCACCTGTGCCA-3' |
| pET28a-ARMC12 Y242F | Forward | 5'-CTGCTGAGCTTCCTGGCACAGAAGAATGACCTTCTCTATGAC-3' |
| | Reverse | 5'-TCTGTGCCAGGAAGCTCAGCAGTCGTACGGCTTGCACCTGTG-3' |
| pET28a-ARMC12 Q245E | Forward | 5'-CTACCTGGCAGAGAAGAATGACCTTCTCTATGACATTCTCAA-3' |
| | Reverse | 5'-GTCATTCTTCTCTGCCAGGTAGCTCAGCAGTCGTACGGCTTG-3' |
| pBiFC-VC155-ARMC12 | Forward | 5'-CCGGAATTCGGATGGGCAAGAGCATCCCCCAATAC-3' |
| | Reverse | 5'-CGGGGTACCTTCCGTGTTTTTAAAGTAGGAACG-3' |
| pBiFC-VN173-MYC | Forward | 5'-ATGCTGGATTTTTTTCGGGTAGTG-3' |
| | Reverse | 5'-CGCACAAGAGTTCCGTAGCTGTTC-3' |
| pEGFP-N1-MYC | Forward | 5'-CCCAAGCTTATGCTGGATTTTTTTCGGGTAGTG-3' |
| | Reverse | 5'-CGCGGATCCCGCACAAGAGTTCCGTAGCTGTTC-3' |
| pEGFP-N1-MYC ∆IDR | Forward-1 | 5'-CCCAAGCTTATGCTGGATTTTTTTCGGGTAGTG-3' |
| | Reverse-1 | 5'-AGGGTGTGACGAGGTCATAGTTCCTGTTGGTGAA-3' |
| | Forward-2 | 5'-CTATGACCTCGTCACACCCTTCTCCCTTCGGGGA-3' |
| | Reverse-2 | 5'-GGTCACGCAGGGCCAGCTTCTCTGAGACGAGCTT-3' |
| | Forward-3 | 5'-GAAGCTGGCCCTGCGTGACCAGATCCCGGAGTTG-3' |
| | Reverse-3 | 5'-CGCGGATCCCGCACAAGAGTTCCGTAGCTGTTC-3' |
| pET28a-EGFP-MYC | Forward | 5'-CCGGAATTCATGCTGGATTTTTTTCGGGT-3' |
| | Reverse | 5'-CCCAAGCTTCGCACAAGAGTTCCGTAGCT-3' |
| pET28a-EGFP-MYC ∆IDR | Forward-1 | 5'-CCGGAATTCATGCTGGATTTTTTTCGGGT-3' |
| | Reverse-1 | 5'-AGGGTGTGACGAGGTCATAGTTCCTGTTGGTGAA-3' |
| | Forward-2 | 5'-CTATGACCTCGTCACACCCTTCTCCCTTCGGGGA-3' |
| | Reverse-2 | 5'-GGTCACGCAGGGCCAGCTTCTCTGAGACGAGCTT-3' |
| | Forward-3 | 5'-GAAGCTGGCCCTGCGTGACCAGATCCCGGAGTTG-3' |
| | Reverse-3 | 5'-CCCAAGCTTCGCACAAGAGTTCCGTAGCT-3' |
| pET28a-mCherry-ARMC12 | Forward | 5'-TGGACAGCAAATGGGTCGCGGAATGGGCAAGAGCATCCCCCA-3' |
| | Reverse | 5'-GCCCTTGCTCACCATGGATCCTTCCGTGTTTTTAAAGTAGG-3' |
| pGL4.1-c-Myc luc | Forward | 5'-CCCACGTGCATAGCACGTGCTATGCACGTGGATACCACGTGGA-3' |
| | Reverse | 5'-CATGGGGTGCACGTATCGTGCACGATACGTGCACCTATGGTGCACCTTCGA-3' |
| pGL3-NUP62 luc | Forward | 5’-CGGGGTACCCAGCCCTTCAGCAATGTTATCCAC-3’ |
| | Reverse | 5’-CCCAAGCTTGCTCCCACCTTCTTTCTTCAGCCG-3’ |
| pGL3-NUP62 luc Mut | Forward | 5’-AGCCAGAGCAAATCAGGGCAGGGAGGGTGGAGGAGGGTAGAGGGG-3’ |
| | Reverse | 5’-CTCCCTGCCCTGATTTGCTCTGGCTCACAGCCCAGCTTCCTTCCT-3’ |
| pGL3-NUP93 luc | Forward | 5’-CGGGGTACCGGGCAGCAGGACAGGGAATGAGTG-3’ |
| | Reverse | 5’-CCCAAGCTTGGGAAGTATGAGCGGCGGGGTTCC-3’ |
| pGL3-NUP93 luc Mut | Forward | 5’-ACCCTTGATATACTACTATTATGGGGATTATAATTCAAGGTGAGATT-3’ |
| | Reverse | 5’-TCCCCATAATAGTAGTATATCAAGGGTGAGACCAGGTGGAGGTAACC-3’ |
| pGL3-NUP98 luc | Forward | 5’-CGGGGTACCGTGGGCCAGTCCCTTAGGGCATCT-3’ |
| | Reverse | 5’-CCCAAGCTTGTGGGGAAGGGGAAGTGTCAGGAG-3’ |
| pGL3-NUP98 luc Mut | Forward | 5’-CTCAGGCTCCGACATCTGTCTGGGCGGGAAGTGCGGGCAGAGAGACT-3’ |
| | Reverse | 5’-CCGCCCAGACAGATGTCGGAGCCTGAGGGCCCTCTTGGAAGGTCTGT-3’ |

ARMC12, armadillo repeat containing 12; NUP62, nucleoporin 62; NUP93, nucleoporin 93; NUP98, nucleoporin 98; Mut, mutataion.

## Table S3 Oligonucleotide sets used for short hairpin RNAs and small guide RNAs

| Primer set | Sequence |
|---|---|
| sh-Scb | 5'-AGGGATACAAGCATATACCACTCGAGTGGTATATGCTTGTATCCCTC-3' (sense);<br>5'-GAGGGATACAAGCATATACCACTCGAGTGGTATATGCTTGTATCCCT-3' (antisense) |
| sh-*ARMC12* #1 | 5’-CCGGTATGACCTTCTCTATGACATTCCTCGAGGAATGTCATAGAGAAGGTCATTTTTTG-3’ (sense);<br>5’-GATCCAAAAAATGACCTTCTCTATGACATTCCTCGAGGAATGTCATAGAGAAGGTCATA-3’ (antisense) |
| sh-*ARMC12* #2 | 5’-CCGGTGGCCATCAAGGCTGGCATAAACTCGAGTTTATGCCAGCCTTGATGGCCTTTTTG-3’ (sense);<br>5’-GATCCAAAAAGGCCATCAAGGCTGGCATAAACTCGAGTTTATGCCAGCCTTGATGGCCA-3’ (antisense) |
| sg*MYC*-a #1 | 5’-CACCGGGAGATCCGGAGCGAATAGGGGG-3’ (sense);<br>5’-AAACCCCCCTATTCGCTCCGGATCTCCC-3’ (antisense) |
| sg*MYC*-a #2 | 5’-CACCGGCTGTAGTAATTCCAGCGAGAGG-3’ (sense);<br>5’-AAACCCTCTCGCTGGAATTACTACAGCC-3’ (antisense) |
| sg*MYC*-i #1 | 5’-CACCGCCTTGCAGCTGCTTAGACGC-3’ (sense);<br>5’-AAACGCGTCTAAGCAGCTGCAAGGC-3’ (antisense) |
| sg*MYC*-i #2 | 5’-CACCGTTTTCGGGTAGTGGAAAACC-3’ (sense);<br>5’-AAACGGTTTTCCACTACCCGAAAAC-3’ (antisense) |

ARMC12, armadillo repeat containing 12.

## Table S4 Identification of MYC-binding proteins by mass spectrometry

| Protein | $log_2FC$ | Protein | $log_2FC$ | Protein | $log_2FC$ |
|---|---|---|---|---|---|
| KDM2A | 8.20059 | NOL11 | 1.92971 | ZC3H13 | 1.31795 |
| PURB | 8.1986 | XRCC1 | 1.9273 | ESF1 | 1.30705 |
| ETV6 | 7.56911 | WDR36 | 1.88225 | NOP58 | 1.305 |
| MIOS | 6.03426 | PARP1 | 1.86231 | HNRNPH3 | 1.2985 |
| TMEM109 | 5.40402 | SNU13 | 1.85505 | CPSF6 | 1.29499 |
| NOP10 | 5.07401 | PTMA | 1.85487 | GTF2E2 | 1.28651 |
| MAX | 4.98669 | NOL10 | 1.85283 | DHX36 | 1.27755 |
| PPIE | 4.73516 | DNTTIP2 | 1.81253 | NCOA5 | 1.27537 |
| GTF2F2 | 4.68745 | SAFB | 1.79753 | PARP2 | 1.27411 |
| PRPF38A | 4.56863 | RBMXL1 | 1.7973 | UTP14A | 1.26528 |
| VIRMA | 4.48085 | PPAN | 1.79305 | UTP3 | 1.25495 |
| CREM | 4.21497 | TBL3 | 1.78914 | HMGN3 | 1.25316 |
| ARMC12 | 4.01901 | UBTF | 1.78144 | CGAS | 1.24841 |
| IMP4 | 4.00298 | WDR3 | 1.772 | DNAJC9 | 1.24617 |
| YTHDC2 | 3.90748 | SEH1L | 1.74661 | GNL2 | 1.24464 |
| PWP1 | 3.89115 | ZC3H11A | 1.73602 | CFAP20 | 1.23541 |
| DDX39A | 3.85322 | RB1 | 1.72604 | DHX9 | 1.22827 |
| NEDD8 | 3.7902 | CDC27 | 1.72353 | CHD1 | 1.219 |
| JADE3 | 3.73508 | NIPBL | 1.72121 | HMG20A | 1.2145 |
| NHP2 | 3.73076 | MAU2 | 1.6974 | TFAP2D | 1.21351 |
| TFAP4 | 3.68096 | UTP15 | 1.68576 | NLE1 | 1.20502 |
| DDX10 | 3.65887 | HNRNPC | 1.66519 | XRCC6 | 1.19855 |
| PSME3IP1 | 3.61664 | NOP2 | 1.66306 | SRSF10 | 1.1944 |
| ZCCHC10 | 3.48588 | LMNA | 1.64491 | RPA3 | 1.18328 |
| CPSF4 | 3.40326 | RBMX | 1.63334 | HMGN1 | 1.17856 |
| VWA5A | 3.21564 | SPOUT1 | 1.62737 | ATAD2B | 1.16898 |
| WDR76 | 3.20909 | RFC1 | 1.62242 | RPA1 | 1.16345 |
| UBP1 | 3.18293 | NOP56 | 1.62058 | PTBP1 | 1.15866 |
| PRPSAP2 | 3.17756 | RPP30 | 1.61559 | MAX | 1.15657 |
| BMS1 | 3.17619 | UTP11 | 1.60724 | EMG1 | 1.15528 |
| HNRNPM | 3.13878 | SMARCA5 | 1.58606 | SRRM1 | 1.15497 |
| HMCES | 3.13066 | RALY | 1.58389 | TOP1 | 1.15036 |
| DDX20 | 3.06295 | ZNF280C | 1.58355 | NOP16 | 1.14694 |
| WDR46 | 3.04008 | SSRP1 | 1.57801 | SNU13 | 1.14426 |
| KRR1 | 2.99692 | DDX27 | 1.57382 | HNRNPA3 | 1.13966 |
| CDK4 | 2.95993 | RSF1 | 1.57147 | EXOSC4 | 1.13942 |
| POLE3 | 2.90998 | AATF | 1.56832 | RBM6 | 1.13031 |
| USP34 | 2.89794 | NOP14 | 1.5642 | AHCTF1 | 1.12925 |
| RIF1 | 2.7932 | BYSL | 1.56143 | HNRNPA1 | 1.12575 |
| RBM28 | 2.73774 | PUM3 | 1.55739 | ILF3 | 1.12244 |
| BAZ1A | 2.71388 | CLASRP | 1.54435 | XRCC5 | 1.1216 |
| DUSP12 | 2.66371 | FBL | 1.50634 | ZC3H14 | 1.12155 |
| NCKIPSD | 2.64937 | KIF1C | 1.50301 | ILF2 | 1.11723 |
| WDR55 | 2.64848 | PDCD11 | 1.49694 | SMCHD1 | 1.11069 |
| OTX1 | 2.61372 | RCL1 | 1.4906 | RRP9 | 1.10873 |
| CENPV | 2.58762 | BAZ2A | 1.48954 | HNRNPL | 1.10475 |
| NAA40 | 2.58025 | INCENP | 1.48893 | RBM12B | 1.10463 |
| DCAF13 | 2.5576 | XPC | 1.48144 | CBX3 | 1.10382 |
| UTP4 | 2.55271 | PFKFB2 | 1.47344 | PTBP3 | 1.10247 |
| RBM42 | 2.54947 | TCF20 | 1.46615 | U2AF2 | 1.09427 |
| LIG3 | 2.52778 | SUPT16H | 1.46306 | MATR3 | 1.09237 |
| TRIM26 | 2.47445 | HP1BP3 | 1.46015 | BRIX1 | 1.085 |
| THOC5 | 2.47174 | RPA2 | 1.45415 | CEBPZ | 1.08487 |
| ZNF326 | 2.46776 | STRBP | 1.45287 | HNRNPH2 | 1.08283 |
| SMC6 | 2.45798 | DRG1 | 1.44441 | SRP14 | 1.08265 |
| CDC73 | 2.41179 | STAU2 | 1.43805 | PTBP2 | 1.08259 |
| MORF4L1 | 2.40773 | GLYR1 | 1.42961 | TFAP2A | 1.0814 |
| APTX | 2.40655 | DIDO1 | 1.42443 | SRSF7 | 1.07972 |
| SAFB2 | 2.3845 | GPATCH4 | 1.42375 | DHX15 | 1.07737 |
| NONO | 2.35717 | PPIG | 1.4129 | TRA2A | 1.06239 |
| RBM15 | 2.32515 | FGF2 | 1.40895 | CBX5 | 1.06185 |
| BAZ1B | 2.27354 | TOP2A | 1.40601 | CHD2 | 1.06068 |
| TDRD3 | 2.26526 | PNKP | 1.4042 | RCC1 | 1.0601 |
| PAK1IP1 | 2.20825 | C1orf131 | 1.40405 | MECP2 | 1.05826 |
| SMARCA1 | 2.17335 | ATRX | 1.40401 | HNRNPA0 | 1.05056 |
| GAR1 | 2.16951 | MSH3 | 1.40336 | HNRNPR | 1.04753 |
| SFPQ | 2.14266 | MPHOSPH10 | 1.39633 | LRRC47 | 1.04169 |
| POLR3F | 2.14252 | H2AC21 | 1.38597 | RRP12 | 1.04064 |
| CUL2 | 2.13922 | FOLR1 | 1.38571 | POLA2 | 1.04007 |
| DNAJB1 | 2.13466 | CHD1L | 1.38083 | BANF1 | 1.03437 |
| NSD2 | 2.12453 | NOL7 | 1.3805 | TFCP2 | 1.02782 |
| ALKBH5 | 2.09317 | IMP3 | 1.38001 | ZEB1 | 1.02623 |
| PAXBP1 | 2.08948 | KAT7 | 1.37179 | MACROH2A1 | 1.01823 |
| UTP18 | 2.08902 | PHIP | 1.36294 | TFIP11 | 1.01619 |
| XIAP | 2.08546 | ZCCHC8 | 1.36212 | SH3BGRL3 | 1.01545 |
| PINX1 | 2.00772 | DDX18 | 1.35404 | WDR82 | 1.01512 |
| RPF2 | 1.99147 | CBX8 | 1.35177 | SAP18 | 1.01286 |
| RPF1 | 1.98017 | H1-10 | 1.3504 | ARAP1 | 1.01215 |
| TMPO | 1.97512 | CHTOP | 1.33736 | MDC1 | 1.00125 |
| RRP7A | 1.96172 | DKC1 | 1.31802 | | |

## Table S5 Identification of target genes associated with outcome of NB

| Up-regulated genes | | Down-regulated genes |
|---|---|---|
| ALDH16A1 | PRDX6 | ALDH3A2 |
| ARFIP2 | PRKCSH | ARHGAP21 |
| ARHGEF2 | PRPF3 | ATP6V1C1 |
| ATP11C | PSMB5 | ATP8A1 |
| ATP13A1 | PTGER2 | BBS9 |
| BAZ1A | REC8 | BEND6 |
| BRCA2 | RECQL4 | C9orf24 |
| BRIP1 | RPUSD2 | CADM1 |
| BRMS1 | SEC13 | CCDC146 |
| BRPF1 | SIAH2 | CLTCL1 |
| CD248 | SLC1A5 | CMAS |
| CDC25A | SLC25A22 | CREBL2 |
| CFL1 | SLC30A3 | DCAF6 |
| CHEK2 | SLC38A5 | DERA |
| CITED1 | SLC41A1 | DLG1 |
| CLMP | SMG8 | EPB41L5 |
| CNOT3 | SNRPB | FAM131B |
| CYP27B1 | SNRPF | FIG4 |
| DAXX | SPATA5L1 | FOXP1 |
| DDR2 | STAMBP | HECTD2 |
| DGAT2 | STK24 | HP1BP3 |
| DNAJB5 | TBX2 | HRK |
| DNTTIP2 | TICAM1 | ICA1L |
| DPF1 | TMEM216 | INPP5K |
| DTL | TMUB1 | KATNAL1 |
| DUSP4 | TNFRSF10B | LRRC8C |
| DYRK3 | TOP1MT | MAGI2 |
| EMILIN1 | TP53I11 | MAP1A |
| FIBP | TRIB3 | MTRF1 |
| FTSJ1 | TRIM28 | MYO9A |
| GLA | YIF1A | PIP5KL1 |
| GTPBP1 | ZNF195 | PPP3CB |
| HMGB2 | ZNF317 | PRKAG1 |
| ING2 | ZNF530 | RAB2A |
| IPO4 | ZNF581 | RHOU |
| KDELR1 | ZNF668 | RORB |
| KLHL25 | | SEC31B |
| LIG1 | | SHF |
| LSM7 | | SLC4A8 |
| LY6E | | SMIM14 |
| MCM5 | | SPOPL |
| MED12 | | SPRYD7 |
| MKRN3 | | SRR |
| MND1 | | SSX2IP |
| MRPL16 | | STX8 |
| MYB | | SYBU |
| NAE1 | | TCTA |
| NFATC3 | | TMCO3 |
| NOB1 | | TMEM107 |
| NUP50 | | TOM1L2 |
| NUP54 | | TRPC1 |
| NUP62 | | TSHZ2 |
| NUP93 | | VPS4B |
| NUP98 | | ZC3H6 |
| PLAGL2 | | |
| PNKP | | |
| PNPT1 | | |
| POLD1 | | |
| PPP1R15B | | |
| PRDX4 | | |

## Table S6 Screening of inhibitors targeting ARMC12-MYC interaction

| MTT colorimetry | | | Dual-luciferase assay |
|---|---|---|---|
| (S)-crizotinib | Duloxetine HCl | Primaquine Diphosphate | Tioconazole |
| Adapalene | Econazole | Proguanil | Aprepitant |
| Afatinib (BIBW2992) | Econazole nitrate | Protriptyline hydrochloride | Auranofin |
| Afatinib (BIBW2992) Dimaleate | Efavirenz | Quetiapine Fumarate | Ceritinib (LDK378) |
| Amiodarone HCl | Elvitegravir (GS-9137, JTK-303) | Quinestrol | Cinacalcet HCl |
| Amitriptyline | Enasidenib Mesylate | Raloxifene | Dasatinib |
| Amlodipine | Erythromycin estolate | Raloxifene HCl | Dihydroergotamine Mesylate |
| Amlodipine Besylate | Estradiol Cypionate | Rotigotine | Erythromycin estolate |
| Amlodipine maleate | Estradiol valerate | Salmeterol | Estradiol valerate |
| Anidulafungin (LY303366) | Ezetimibe | Salmeterol Xinafoate | Flibanserin |
| Aprepitant | Fedratinib (SAR302503, TG101348) | Sertaconazole nitrate | Grazoprevir |
| Asenapine maleate | Felodipine | Sertraline HCl | Lovastatin |
| Auranofin | Fingolimod | Simeprevir | Menadione |
| Azelastine HCl | Flibanserin | Simvastatin | Methylene Blue |
| BAF312 (Siponimod) | Fluoxetine HCl | Sulconazole Nitrate | Nefazodone hydrochloride |
| Bazedoxifene Acetate | Fluphenazine dihydrochloride | Sunitinib Malate | Quinestrol |
| Bazedoxifene HCl | Gefitinib (ZD1839) | Tacrolimus (FK506) | Raloxifene |
| Bepridil hydrochloride | Grazoprevir | Tafluprost | Rotigotine |
| Betamethasone Valerate | Hydroxyzine 2HCl | Tamoxifen | Tamoxifen Citrate |
| Brexpiprazole | Ibrutinib (PCI-32765) | Tamoxifen Citrate | Trifluoperazine |
| Butoconazole | Indacaterol | Tegaserod Maleate | |
| Butoconazole nitrate | Indacaterol Maleate | Telotristat Etiprate (LX 1606 Hippurate) | |
| Calcipotriene | Ivacaftor (VX-770) | Terconazole | |
| Calcitriol | Ivermectin | Tezacaftor?(VX-661) | |
| Canagliflozin | Lansoprazole | Thioridazine hydrochloride | |
| Canagliflozin hemihydrate | Latanoprost | Thiothixene | |
| Candesartan Cilexetil | Lomitapide | Ticagrelor | |
| Carvedilol | Loperamide HCl | Tioconazole | |
| Carvedilol Phosphate | Loratadine | Toremifene Citrate | |
| Celecoxib | Lovastatin | Travoprost | |
| Ceritinib (LDK378) | Luliconazole | Triclosan | |
| Ceritinib dihydrochloride | Lusutrombopag | Trifluoperazine | |
| Chlorhexidine | Maprotiline HCl | Trifluoperazine 2HCl | |
| Chlorhexidine?2HCl | Mefloquine HCl | Triflupromazine hydrochloride | |
| Chloroxine | Menadione | Vilanterol Trifenate | |
| Chlorpromazine | Methylene Blue | Vilazodone | |
| Chlorpromazine HCl | Miconazole | Vinorelbine Tartrate | |
| Chlorprothixene | Miconazole Nitrate | Vortioxetine | |
| Cinacalcet | Mitotane | Vortioxetine (Lu AA21004) HBr | |
| Cinacalcet HCl | Montelukast | | |
| Clemastine Fumarate | Montelukast Sodium | | |
| Clioquinol | Moxidectin | | |
| Clomipramine HCl | Nebivolol | | |
| Clotrimazole | Nebivolol HCl | | |
| Cobicistat (GS-9350) | Nefazodone hydrochloride | | |
| Crizotinib (PF-02341066) | Netupitant | | |
| Cyproheptadine hydrochloride | Nisoldipine | | |
| Dacomitinib (PF299804, PF299) | Nortriptyline hydrochloride | | |
| Dasatinib | Osimertinib (AZD9291) | | |
| Dasatinib hydrochloride | Osimertinib mesylate | | |
| Desipramine Hydrochloride | Ospemifene | | |
| Desogestrel | Oxybutynin | | |
| Dienestrol | Ozanimod (RPC1063) | | |
| Dihydroergotamine Mesylate | Paroxetine HCl | | |
| Doxazosin | Paroxetine mesylate | | |
| Doxazosin Mesylate | Penbutolol Sulfate | | |
| Doxercalciferol | Perphenazine | | |
| Dronedarone | Pimavanserin | | |
| Dronedarone HCl | Pimozide | | |
| Duloxetine | Ponatinib (AP24534) | | |

## Table S7 Identification of TCZ-binding proteins by mass spectrometry

| Protein | $log_2FC$ |
|---|---|
| ABHD11 | 3.61977528 |
| ACAD10 | 2.69059883 |
| ACSL6 | 7.73759139 |
| ADD3 | 2.77414007 |
| AKTIP | 2.65347884 |
| ALAS1 | 3.30379115 |
| ALKBH2 | 3.11270156 |
| ANGEL2 | 1.90907936 |
| ANKRD11 | 3.77849481 |
| AP3M2 | 2.45576453 |
| APOO | 1.14847122 |
| ARMC12 | 6.98867069 |
| ATAD2 | 1.81283811 |
| ATG12 | 3.24571524 |
| ATPAF2 | 3.93107175 |
| BAZ2A | 1.35701902 |
| BBX | 4.91136001 |
| BPGM | 4.7184945 |
| CC2D1B | 2.66651895 |
| CDC23 | 1.50112165 |
| CENPU | 2.09363868 |
| CLCC1 | 6.30074306 |
| CLN5 | 3.9068348 |
| COA3 | 4.30543312 |
| COIL | 1.83854219 |
| COL18A1 | 5.93242669 |
| COX6A1 | 2.83086337 |
| CRAT | 1.13683865 |
| CROT | 3.07879794 |
| CSNK1G3 | 1.35280752 |
| CSPG4 | 2.29070123 |
| CTSA | 1.17390481 |
| DACH1 | 4.24935427 |
| DBR1 | 4.33421605 |
| DEPDC1B | 1.95028045 |
| DES | 5.50358618 |
| DHODH | 5.75573114 |
| DOCK1 | 1.20595593 |
| DPCD | 2.83690443 |
| DPF2 | 6.29014061 |
| DPP9 | 1.8418003 |
| EHBP1L1 | 1.74516404 |
| ENDOV | 1.33046354 |
| ENPP4 | 2.56307604 |
| ENPP7 | 4.14113559 |
| EPHA2 | 1.13255485 |
| ERO1B | 4.6645248 |
| EXOC1 | 2.6758868 |
| F8A1 | 2.54994637 |
| FAM13B | 1.52335924 |
| FASTKD2 | 4.95410854 |
| FASTKD5 | 4.88877669 |
| FBXL18 | 2.44539299 |
| FECH | 4.66739734 |
| GAMT | 4.79210405 |
| GLDC | 5.84761285 |
| GRPEL2 | 3.29615985 |
| GTF2E1 | 4.32612731 |
| H3C1 | 2.25378219 |
| HAUS6 | 5.22934627 |
| HDAC3 | 3.81930609 |
| HSPA2 | 3.96116039 |
| IFT70B | 1.89012137 |
| IKBIP | 4.90546218 |
| IMP4 | 1.2950141 |
| IST1 | 5.9264137 |
| KATNA1 | 3.08550182 |
| KCTD1 | 2.34865574 |
| KXD1 | 2.92401997 |
| KYAT1 | 4.04208613 |
| LAMTOR2 | 1.32471535 |
| LBH | 4.03744402 |
| LDB1 | 4.3099737 |
| LGALS3BP | 3.85301745 |
| LIN37 | 1.99204048 |
| MBIP | 3.97089556 |
| MEAK7 | 2.27618524 |
| MECR | 3.93614069 |
| MRC2 | 3.50182415 |
| MRPL20 | 2.00333717 |
| MRPL51 | 4.41355784 |
| MRPS14 | 4.3190597 |
| MT-ATP6 | 5.44737132 |
| MTFR1L | 1.75782611 |
| MTG1 | 1.69780896 |
| MTIF2 | 3.2860458 |
| MT-ND4 | 1.99556276 |
| NBEAL2 | 1.68014173 |
| NDUFAF6 | 3.45496546 |
| NDUFS5 | 5.28094921 |
| NDUFS7 | 5.03010422 |
| NEBL | 3.70231603 |
| NEMF | 2.72742317 |
| NHERF2 | 3.46409028 |
| NME4 | 2.37170434 |
| NOP10 | 3.86176969 |
| NSMCE1 | 1.39330276 |
| NT5DC3 | 4.91580891 |
| NTHL1 | 2.53575404 |
| OTUB2 | 2.89586886 |
| OXSM | 4.92887835 |
| P3H3 | 4.5777604 |
| PACSIN3 | 6.05560506 |
| PAK4 | 2.5662295 |
| PFKFB2 | 2.78809548 |
| PGS1 | 2.35550688 |
| PIK3R2 | 2.75 |
| PISD | 5.07834637 |
| PLG | 2.4630962 |
| POLR1H | 1.07745908 |
| PPP2R5E | 3.48856561 |
| PRAF2 | 2.52030053 |
| PRKCD | 4.64020128 |
| PTDSS1 | 2.94975976 |
| PTGR3 | 4.58946427 |
| PYGM | 5.13126199 |
| RAB3D | 3.2316429 |
| RARS2 | 2.9558212 |
| RASAL2 | 1.64130982 |
| RMND5A | 4.36925657 |
| SELENOT | 4.76405594 |
| SLC25A12 | 1.98994761 |
| SLC4A7 | 3.19712145 |
| SLIT1 | 5.3538743 |
| SMAP1 | 3.33809543 |
| SMC5 | 1.146668 |
| SMC6 | 4.85099336 |
| SNCG | 4.18367181 |
| SNX8 | 3.41991804 |
| SPCS3 | 3.49222769 |
| SPOUT1 | 1.14085927 |
| SRR | 3.59059324 |
| STAT2 | 1.44108918 |
| STX10 | 3.98002118 |
| STX4 | 3.67299653 |
| SUCLA2 | 3.54814372 |
| TAF2 | 2.25448522 |
| TIMM8B | 3.52274964 |
| TMEM205 | 6.80145432 |
| TRAPPC5 | 1.61723212 |
| TRMT2B | 2.5396186 |
| UQCC1 | 3.11094234 |
| XRCC4 | 3.04459332 |
| YBEY | 4.1041352 |
| ZNF512 | 2.50829678 |
| ZNG1F | 2.36477878 |

## Table S8 18S rRNA analysis of NB and GNB tissues

| Phylum | GNB-Tissue | NB-Tissue |
|---|---|---|
| Ascomycota | 0.102826562 | 1.074615472 |
| Basidiomycota | 0.064266601 | 0.414811699 |
| Cercozoa | 0.004284440 | 0.061929634 |
| Gracilipodida | 0.000000000 | 0.031549059 |
| Phragmoplastophyt | 0.271477703 | 0.847929642 |
| Vertebrata | 99.55130228 | 97.46984338 |

| Genus | NB-Tissue | GNB-Tissue |
|---|---|---|
| Aspergillus | 0.007789891 | 0.000000000 |
| Cercomonadidae | 0.000000000 | 0.000389495 |
| Cladosporium | 0.075561943 | 0.017527255 |
| Filobasidium | 0.000000000 | 0.001168484 |
| Gracilipodida | 0.031549059 | 0.000000000 |
| Magnoliophyta | 0.832739354 | 0.090362736 |
| Malassezia | 0.153850348 | 0.046739346 |
| Mammalia | 97.46984338 | 99.55130228 |
| Naganishia | 0.006621407 | 0.000000000 |
| Pinophyta | 0.015190288 | 0.181114967 |
| Preussia | 0.001557978 | 0.000000000 |
| Rhodotorula | 0.207990091 | 0.000000000 |
| Saccharomyces | 0.706932614 | 0.031549059 |
| Schizophyllum | 0.042065412 | 0.000000000 |
| Talaromyces | 0.017916749 | 0.000778989 |

## Table S9 Identification of UU-T02-binding proteins by mass spectrometry

| Protein | log$_2$FC |
|---|---|
| ABHD11 | 3.21231979 |
| ACAD10 | 3.23832264 |
| ALAS1 | 4.27644756 |
| ALG12 | 2.3117023 |
| ALG6 | 2.26013307 |
| ALKBH2 | 2.85252474 |
| ANGEL2 | 1.98590377 |
| ANKRD11 | 4.29730618 |
| ANKRD28 | 2.44511034 |
| AP3M2 | 2.36262245 |
| APOO | 1.47990832 |
| APOOL | 5.72863524 |
| ARMC12 | 8.0264864 |
| ATAD2 | 1.69443586 |
| ATG12 | 2.47868509 |
| ATPAF2 | 3.85863233 |
| BAZ2A | 2.46576708 |
| BCKDHA | 1.19230278 |
| BPGM | 4.39600753 |
| CAMK1 | 2.47360161 |
| CC2D1B | 2.48701234 |
| CCDC12 | 5.88046924 |
| CDC23 | 2.03615016 |
| CENPO | 5.14091661 |
| CENPU | 2.50717112 |
| CLCC1 | 6.14823119 |
| CLN5 | 3.50458307 |
| COA3 | 5.642247 |
| COL18A1 | 5.69017535 |
| COMTD1 | 2.66436325 |
| COX6A1 | 3.96275427 |
| COX6C | 1.54478862 |
| CROT | 2.78979802 |
| CSNK1G3 | 1.74890959 |
| CSPG4 | 2.39125932 |
| CTSA | 1.5319868 |
| CYB561D2 | 1.4596457 |
| DACH1 | 3.61186517 |
| DBR1 | 2.50218178 |
| DES | 5.51612288 |
| DHODH | 6.12605364 |
| DMAC2L | 1.64760908 |
| DNAJB4 | 1.67004152 |
| DPCD | 2.67179471 |
| DPP9 | 2.19141966 |
| DUS2 | 4.05070768 |
| EHBP1L1 | 2.50255223 |
| EIF2AK4 | 1.16906094 |
| ENPP7 | 4.49153498 |
| EPHA2 | 1.14371158 |
| ERCC3 | 1.52315011 |
| ERI3 | 4.59906514 |
| ERO1B | 3.82760374 |
| EXOC1 | 3.38308782 |
| F8A1 | 3.11387646 |
| FASTKD2 | 4.92188523 |
| FASTKD5 | 4.76577281 |
| FBXL18 | 2.38584979 |
| FECH | 4.92759028 |
| GAMT | 4.50194434 |
| GHITM | 1.93142723 |
| GLDC | 5.89833719 |
| GLE1 | 3.0895287 |
| GMPR | 2.79976134 |
| GRPEL2 | 3.53568925 |
| GTF2E1 | 4.22807181 |
| H3C1 | 2.80677214 |
| HAUS6 | 5.01495778 |
| IFT70B | 2.36881027 |
| IGF1R | 1.35363961 |
| IKBIP | 4.92548889 |
| IMP4 | 1.31215196 |
| IST1 | 6.06454863 |
| KATNA1 | 3.11148346 |
| KXD1 | 2.79315238 |
| KYAT1 | 3.76570588 |
| LAMTOR2 | 2.58091912 |
| LBH | 3.47462215 |
| LDB1 | 4.12446135 |
| LGALS3BP | 4.5702896 |
| LIN37 | 1.70257 |
| LMBR1L | 1.17633 |
| LRATD2 | 4.52896 |
| MBIP | 3.60028 |
| MBLAC2 | 1.45482 |
| MEAK7 | 2.21353 |
| MECR | 4.75746 |
| MIOS | 2.83367 |
| MLYCD | 3.66279 |
| MPRIP | 4.34749 |
| MRC2 | 3.84443 |
| MRPL16 | 1.68968 |
| MRPL20 | 1.56476 |
| MRPL51 | 4.77093 |
| MRPL57 | 3.54151 |
| MRPS14 | 4.07925 |
| MT-ATP6 | 5.13675 |
| MTFR1L | 1.32741 |
| MTG1 | 2.45602 |
| MTIF2 | 3.83324 |
| MT-ND4 | 2.34435 |
| NBEAL2 | 1.15466 |
| NDUFAF6 | 3.45196 |
| NDUFS7 | 5.18499 |
| NEBL | 4.40341 |
| NEMF | 2.73708 |
| NHERF2 | 2.95744 |
| NME4 | 2.90275 |
| NNT | 4.45715 |
| NOP10 | 4.43032 |
| NOTCH2 | 3.5928 |
| NR3C1 | 4.62953 |
| NSMCE1 | 1.85243 |
| NT5DC3 | 4.37947 |
| NTHL1 | 2.53012 |
| OSGEPL1 | 2.48379 |
| OXNAD1 | 4.49696 |
| OXSM | 5.07642 |
| P3H3 | 4.62992 |
| PACSIN3 | 6.02634 |
| PAK4 | 1.75839 |
| PCYOX1L | 4.62911 |
| PFKFB2 | 3.14599 |
| PGS1 | 1.63619 |
| PIK3R2 | 2.92696 |
| PIP4P1 | 1.09049 |
| PISD | 5.31647 |
| POLG | 2.08938 |
| PPP1R12C | 1.30518 |
| PPP6R2 | 1.82203 |
| PRAF2 | 2.48638 |
| PRKAA2 | 3.23091 |
| PRKCD | 4.61064 |
| PTDSS1 | 3.34638 |
| PTGR3 | 4.50574 |
| PYGM | 5.06577 |
| R3HCC1 | 3.71893 |
| RAB34 | 3.61034 |
| RAB3D | 2.74721 |
| RAB8B | 2.05056 |
| RARS2 | 2.89749 |
| RBM6 | 1.82996 |
| RMND5A | 4.73425 |
| RREB1 | 1.3333 |
| RSAD1 | 1.00651 |
| SAR1B | 1.72209 |
| SEL1L | 2.90183 |
| SELENOT | 4.87032 |
| SLC12A7 | 2.50293 |
| SLC25A12 | 2.70947 |
| SLC4A7 | 3.16733 |
| SLIT1 | 4.97154 |
| SMC5 | 1.54782 |
| SMC6 | 4.85952 |
| SNCG | 3.63078 |
| SNF8 | 1.79373 |
| SPOUT1 | 2.37465 |
| SRR | 3.66231 |
| STAT2 | 2.53868 |
| STK3 | 3.30788 |
| STX10 | 4.07272641 |
| STX4 | 3.92041893 |
| SUCLA2 | 3.81367739 |
| SUN1 | 6.67942939 |
| TAF2 | 2.3696954 |
| TAF9B | 1.21062407 |
| TATDN3 | 1.00305677 |
| TMED7 | 2.32572033 |
| TMEM205 | 7.64855216 |
| TMEM256 | 6.48984145 |
| TRAPPC5 | 1.35306168 |
| TRIT1 | 3.00090694 |
| TRMT2B | 2.11117794 |
| TTC28 | 1.61987678 |
| TXNL4A | 6.00147199 |
| UACA | 4.05988216 |
| UNC79 | 3.55026257 |
| UQCC1 | 3.24961987 |
| YBEY | 5.66401543 |
| ZBTB7A | 3.64744044 |
| ZCCHC9 | 3.24670526 |
| ZNF512 | 3.38993044 |
| ZNG1F | 2.11577998 |
